# Implementation of digital tuberculosis information systems: perspectives from 10 high TB burden countries

**DOI:** 10.1186/s12879-026-12852-3

**Published:** 2026-04-02

**Authors:** Charity Oga-Omenka, Joel Shyam Klinton, Darryl Ku, Petra Heitkamp, Aderonke Vivian Agbaje, Plinio P. Morita, Warren Dodd

**Affiliations:** 1 School of Public Health Sciences, University of Waterloo, Waterloo, ON Canada; 2 TB-PPM Learning Network, McGill University Health Centre – Research Institute, Montreal, QC Canada; 3 McGill International TB Centre, McGill University, Montreal, QC Canada; 4 Institute of Human Virology, Abuja, Nigeria; 5 Department of Systems Design Engineering, University of Waterloo, Waterloo, Canada; 6 Centre for Digital Therapeutics, Toronto General Hospital Research Institute, University Health Network, Toronto, ON Canada; 7 Université de technologie de Compiègne, CNRS, Biomechanics and Bioengineering, UMR CNRS 7338, Compiègne, France

**Keywords:** Tuberculosis, Digital health, Implementation, Information systems, Global health, Technology adoption

## Abstract

Tuberculosis (TB) remains a major global health crisis, with 10.8 million people diagnosed annually and 2.7 million cases going undetected in 2023. While TB digital health information systems offer promising solutions for detection and management in low- and middle-income countries, implementation effectiveness varies significantly across health system contexts. However, no studies have systematically examined implementation experiences across multiple high-burden countries using implementation science frameworks, leaving National TB Program (NTP) managers without evidence-based guidance for context-appropriate system selection and deployment strategies. We conducted semi-structured interviews with 15 TB program partners across 10 high TB burden countries to evaluate digital health system implementation in national TB programs. Semi-structured interviews were used. The data was analyzed using the Consolidated Framework for Implementation Research (CFIR). Systems were assessed for advantages, disadvantages, and implementation challenges, with findings organized across all five CFIR domains to identify policy-relevant implementation success factors. Across the countries, 29 distinct TB information systems were documented with varying implementation success. Key advantages included real-time data availability, patient-level tracking, and care cascade monitoring. Primary disadvantages included internet dependency, limited interoperability, and parallel paper-based requirements. Implementation success was determined by five policy-relevant factors: innovation adaptability, external policy drivers, organizational readiness (health system infrastructure availability), individual champions (tech-savvy staff), and implementation processes. For health policymakers and NTP managers, successful digital TB system implementation requires coordination across key implementation areas rather than technological sophistication alone. Countries should establish collaborative governance frameworks, prioritize contextual adaptation with offline capabilities, cultivate technical champions, and adopt user-centered design approaches. The study provides a practical framework for NTP managers to assess implementation readiness and develop context-appropriate digital health strategies that support health systems strengthening in low- and middle-income countries.

**Supplementary Information:**

The online version contains supplementary material available at 10.1186/s12879-026-12852-3.

## Key Messages

Digital tuberculosis (TB) information systems are increasingly being adopted in countries with a high TB burden; however, their successful implementation relies more on the readiness of health systems and alignment with local contexts than on the complexity of the technology itself.

Evidence from ten high-burden TB countries indicates that effective implementation hinges on five interrelated factors: adaptability of innovations, supportive policy frameworks, organizational preparedness, usercompetency, and well-structured implementation strategies.

Countries that exhibit robust governance, coordinated partnerships, and ongoing domestic funding tend to deliver more reliable data quality andadoption, while those that depend heavily on donor funding or fragmented systems face challenges in scalability.

For policymakers and managers of National TB Programs (NTPs), prioritizing assessments of implementation readiness, ensuring offline capabilities, focusing on user-centered design, enhancing capacity, and establishing sustainable financing models are crucial steps to ensure that digital TB systems bolster health infrastructures and aid in achieving TB elimination objectives.

**Supplementary Information:**

The online version contains supplementary material available at 10.1186/s12879-026-12852-3.

## Background

The World Health Organization (WHO) has emphasized the crucial role of effective monitoring and surveillance systems in combating tuberculosis (TB), given that it is a major global health crisis [1]. As of 2023, over 10 million people are diagnosed with TB each year, with nearly 3 million cases going undetected, revealing critical weaknesses in global TB management [1]. According to the WHO’s 2024 Global TB report, these 10 high burden countries accounted for 64% of the global burden: India (26% with approximately 2,8 million cases), Indonesia (10% with 1.1 million cases), the Philippines (6.8% with about 739,000 cases), Pakistan (6.4% with 686,000 cases), Nigeria (4.6% with 499,000 cases), Bangladesh (3.5% with 379,000 cases), Myanmar (2.8% with 302,000 cases), Vietnam (1.7% with 182,000 cases), Kenya (1.1% with 124,000 cases), and Tanzania (1.1% with about 122,000 cases) [1]. These same 10 countries also accounted for an estimated 61% of the world’s missing TB cases—those not diagnosed or reported—totaling over 1.58 million people, including India (400,000), Indonesia (295,200), Pakistan (210,200), Myanmar (172,900), the Philippines (163,200), Nigeria (131,700), Vietnam (77,500), Bangladesh (75,200), Tanzania (29,600), and Kenya (29,300) (1).

TB information systems are digital platforms designed to capture, store, manage, and transmit data across the TB care cascade, including case notification, patient registration, treatment monitoring, laboratory results management, and surveillance reporting. Unlike paper-based registers that have historically served these functions, digital TB information systems enable real-time data access, automated reporting, patient-level tracking, and integration across multiple points of care. Effective TB surveillance systems require several key characteristics: timely case detection and notification, complete and accurate patient-level data, interoperability with laboratory and pharmacy systems, accessibility for both public and private sector providers, and the capacity to generate actionable insights for program management [4–9]. However, transitioning from paper-based to digital systems presents substantial implementation challenges, particularly in low- and middle-income countries (LMICs) where National TB Programs (NTPs) must navigate complex decisions about technology selection, deployment strategies, and sustainability without evidence-based guidance on context-appropriate approaches [4–6, 10].

Despite the proliferation of digital TB information systems globally, a critical knowledge gap persists regarding how these systems perform in real-world implementation across diverse health system contexts. While paper-based systems are plagued by issues such as poor data accuracy, illegibility, incomplete records, and delays in reporting that compromise care quality and hinder program planning [11–15], the transition to digital systems introduces new challenges. Health policymakers and NTP managers face complex decisions about balancing technological capabilities with local infrastructure constraints (connectivity, power supply, hardware availability), health workforce capacity (digital literacy, training requirements, staff continuity), and long-term sustainability considerations (ongoing costs, technical support, donor dependency) [9, 10, 16–19]. Existing research often focuses on single-country experiences or specific technological solutions [20–26], failing to systematically examine implementation experiences across multiple high-burden countries using implementation science frameworks [27]. This leaves NTP managers without comparative evidence to guide context-appropriate system selection and deployment strategies.

**Fig. 1:**
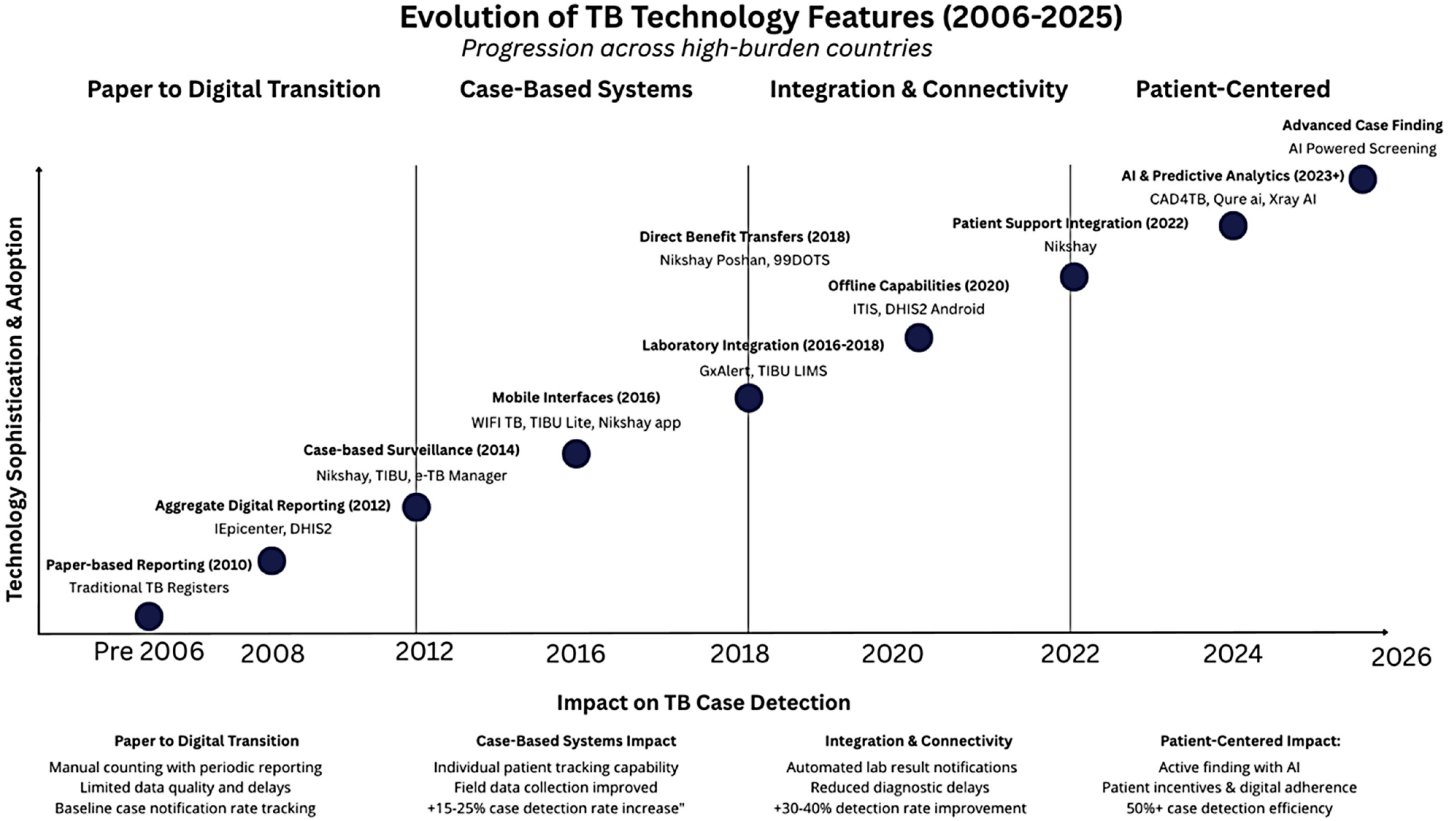
Evolution of TB technology features (2006– 2025+)

In response to limitations of conventional approaches, many high burden countries transitioned from paper-based methods to digital health information systems, beginning with aggregate digital reporting systems around 2008 and progressing to case-based surveillance by 2012 [7, 11–15]. This evolution has continued through several phases (Fig. 1), with mobile interfaces emerging around 2016, laboratory integration systems between 2016 and 2018 automating result notifications, and offline capabilities becoming standard features by 2020 to address connectivity challenges. Countries such as India, Indonesia, the Philippines, Kenya, and others have deployed diverse digital TB information systems—ranging from comprehensive nationwide platforms to specialized applications addressing specific components of the TB care cascade [14, 28].

This evolution continued with mobile interfaces emerging around 2016, followed by laboratory integration systems between 2016 and 2018, which automated result notifications and reduced diagnostic delays [29, 30]. For instance, Nikshay (India’s national TB information system) facilitates patient tracking [3], while Indonesia’s SITB (Sistem Informasi Tuberkulosis/TB Information System) enables real-time collection of patient data [31]. Mobile health applications in Nigeria and Kenya further empower healthcare workers to deliver care in remote areas, ensuring timely and effective treatment for more patients [32–34].

By 2020, the evolution continued with offline capabilities becoming standard features in many TB digital systems, addressing connectivity challenges in remote areas [35–37]. These apps facilitate timely follow-ups, ensure treatment adherence, and improve communication between patients and healthcare providers [14, 32, 33, 37].

The shift towards digital solutions addresses many limitations of traditional paper-based methods. Paper-based systems are often plagued by issues such as poor data accuracy, illegibility, filing and incomplete records, which compromise care quality and hinder effective planning [38, 39]. Digital systems can mitigate these problems by offering rapid and accurate diagnostics, facilitating real-time data access, automating data management, and supporting remote patient monitoring^21^. Looking ahead to 2026 and beyond, advanced case finding intelligence is expected to transform TB care further with predictive algorithms for targeting high-risk populations and smart resource allocation [40–45]. This transition not only improves the effectiveness of TB programs but also enhances patient outcomes through timely and precise data.

Despite technological advancements, a crucial knowledge gap persists regarding how digital TB systems integrate with existing health technologies and impact patient care in real-world settings. Existing research often fails to account for practical implementation difficulties [46], limiting comprehensive understanding of system effectiveness and compatibility with other health technologies [14, 47].

This qualitative study assessed technology-based TB control systems through semi-structured interviews with implementers from 10 high-burden countries, examining [1]: available services [4]; implementation enablers [5]; adoption barriers; and [6] integration opportunities. Understanding these factors is essential for scaling effective digital health solutions that can potentially impact nearly two-thirds of the global TB burden.

## Methodology

### Study design

We employed a qualitative descriptive approach (Sandelowski, 2000) to explore the implementation of TB information systems across 10 high-burden countries. This design enabled us to remain closely aligned with respondents’ narratives and authentically reflect the nuanced realities of implementing TB information systems.

### Conceptual framework

**Fig. 2:**
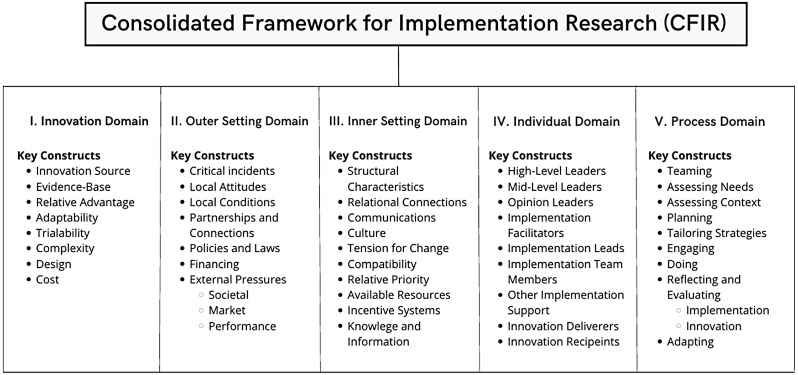
Consolidated framework for implementation research (CFIR)

We used the updated 2022 Consolidated Framework for Implementation Research (CFIR) [1] to guide our study design, data collection, and analysis. We selected CFIR for three reasons [1]: its structure addresses multiple levels of implementation influence, essential for understanding complex health information systems [4]; its established use in global health settings allows for systematic comparison of findings; and [5] its domains specifically capture technological attributes alongside contextual factors critical for digital health implementation. This framework comprises five domains with 43 constructs: Innovation Characteristics (8 constructs), Outer Setting (7 constructs), Inner Setting (10 constructs), Characteristics of Individuals (9 constructs), and Implementation Process (9 constructs) (Fig. 2 and Appendix A) [48].

### Participant selection

In collaboration with the TB Public-Private Mix Learning Network (TBPPM LN), 10 countries were selected for their high TB prevalence and significant private sector involvement. These countries represent diverse health system contexts across Asia and Africa: Bangladesh, Tanzania, Indonesia, India, Pakistan, Nigeria, Myanmar, the Philippines, Kenya, and Vietnam. The 10 countries were selected based on three criteria [1]: classification as high TB burden countries by WHO (among top 30 globally) [4]; documented private sector involvement in TB care delivery (≥30% of cases detected by private providers) [49]; and [5] existence of at least one operational digital TB information system. We applied purposive sampling to recruit key policy implementers and program managers involved in digital TB system deployment in these countries. To ensure diverse perspectives, we initially contacted two program implementers from each country, representing both the national TB program and the private sector implementation partners. Inclusion criteria included [1] direct involvement in TB digital health system implementation or management for ≥6 months [4]; working knowledge of the system’s technical features and deployment context; and [5] ability to speak about implementation challenges and facilitators. Of the 30 implementers contacted, 15 agreed to participate as key informants, with at least one representative from each of the selected countries providing insights into their national digital health implementation experiences. The unit of analysis was each distinct TB information system including any digital platform for TB case notification, patient tracking, treatment monitoring, or laboratory management operational during data collection (October 2023-February 2024), including both national systems and regional pilots.

### Data collection

Semi-structured interviews were conducted to obtain in-depth insights into respondents’ experiences with national TB information systems from a health systems implementation perspective. The interview guide, developed using the updated 2022 CFIR, structured these policy-focused discussions. We conducted the interviews remotely via Zoom from October 2023 to February 2024. Interviews were conducted using an interview guide developed specifically for this study (see Appendix D). The guide explored healthcare providers’ perspectives and experiences with technology use across the TB care cascade. All interviews (*n* = 15) were conducted remotely via Zoom with verbal consent obtained and documented in audio recordings, following protocols approved by our institutional ethics board for minimal-risk interview research. Interview recordings were transcribed verbatim for detailed analysis. Interviews lasted 45–75 minutes, conducted in English by the first and second authors. All participants were fluent in English. Audio recordings were transcribed verbatim by a professional service and reviewed for accuracy by the interviewers.

### Data analyses

We analyzed the data using the iterative framework method developed by Richie and Spencer (2002) [50] and adapted by Gale et al. (2013) [51] for applied health research. This method involves seven key stages: transcription, familiarization with the data, coding, developing an analytical framework, applying the framework, charting data into the framework matrix, and interpreting the data.

We enhanced the framework method by integrating an inductive-deductive analytical approach. Initially, one author performed inductive coding on the first five transcripts, developing an initial codebook. This codebook was then iteratively refined through several reviews and discussions. Another author categorized and organized the coded data according to the CFIR framework, reflecting respondents’ experiences, supervised by the two authors, to further refine and validate the categories and themes. The team used NVivo 12 to manage and organize the coding process.

Subsequently, the first author conducted two complementary analyses [1]: a structured assessment of technological advantages, disadvantages, and implementation challenges across all systems, and [4] a comprehensive cross-country analysis using the CFIR framework to identify patterns in implementation factors. This dual analytical approach enabled both a practical evaluation of TB technologies and a theoretically grounded understanding of implementation contexts. Final themes were validated through team consultation to ensure analytical rigour while preserving country-specific nuances.

TB technology system functionality and implementation status were primarily assessed through semi-structured interviews with TB program implementers. While implementation timelines were cross-referenced with Stop TB Partnership documentation [52] and internet searches, interview data took precedence where discrepancies existed, as official reports may not reflect current operational status.

## Results

### Demographic details

Our participants included 15 implementers across sectors and from 10 countries reflecting a range of ages, educational backgrounds, and professional affiliations. Ten participants were male, 9 from the private sector, and 12 had graduate degrees. Participants included a mix of non-governmental organizations (NGOs) and for-profit organization representatives, with notable public sector involvement in Indonesia and India. Most were between 35 and 50 years old, representing countries such as Tanzania, Kenya, Bangladesh, Nigeria, India, and the Philippines—suggesting a depth of professional experience. A smaller group from Indonesia and Myanmar, aged 25 to 35, added generational diversity. Educational qualifications were generally high, with most holding Master’s degrees and several possessing doctorates. Their demographic details are outlined below in Table 1.

**Table 1:** Participants country and sector representation

Country, Participant ID	Gender	Sector	Education	Position/Context	Experience
**Bangladesh,** T1006	Male	Private	Masters	Senior TB program management at NGO	~10 years in TB work
**India,** T1009	Male	Public	Masters	TB epidemiology specialist at international organization	Multiple years in international development
**India,** T1010	Female	Private	Masters	Health director at implementing NGO	11+ years in TB work
**Indonesia,** T1002	Male	Public	Professional Masters	Mid-level management at health NGO	Not specified
**Kenya,** T1014	Male	Public	Masters	M&E coordination with statistical background	Multiple years with NTP
**Kenya,** T1005	Male	Private	Bachelors	Technical advisor for TB programs	~7 years (since 2016)
**Myanmar,** T1008	Male	Private	Masters	Management position at health NGO	Not specified
**Nigeria,** T1007	Male	Private	Doctoral	M&E and surveillance specialist	~1 year in current role
**Nigeria,** T1015	Male	Public	Masters	Senior State TB program management	10+ years in TB work
**Pakistan,** T1011	Female	Private	Masters	TB program consultant, formerly in management	10+ years in TB work
**Philippines,** T1012	Male	Public	Bachelors	IT implementation specialist	Multiple years with system development
**Philippines,** T1013	Female	Private	Professional Masters	Senior management for TB innovation project	Since 2018 in current project
**Tanzania,** T1003	Female	Public	Masters	Government TB program staff	Not clearly specified
**Tanzania,** T1001	Female	Private	Masters	M&E leadership at local NGO	~7 years (since 2016)
**Vietnam,** T1004	Male	Private	Bachelors	Information systems management at international NGO	Not specified

### Overview of TB technologies by country

The assessment of TB information systems revealed diverse technological approaches across the ten high-burden countries (Table 2, Appendix B), ranging from nationwide platforms to specialized applications addressing specific components of the TB care cascade.

**Table 2:** Summary of TB technology implementation challenges

Country	Primary System(s)	Key Features	Main Implementation Challenges
Bangladesh	e-TB Manager, Janao app, TBCP	• Digital patient database • Mobile notification for private sector	• Limited access (lab technicians only) • No offline capabilities • Urban-rural digital divide
India	Nikshay ecosystem, Epicenter	• Comprehensive case-based surveillance • Direct benefit transfers • Supply chain integration	• Connectivity in rural areas • Dual reporting requirements • Variable technical literacy
Indonesia	SITB (2022)	• Real-time TB information • Specialized apps for different functions • Mobile solutions	• Server capacity limitations • Complex interfaces • Ownership transitions
Kenya	TIBU system	• Case-based data since 2012 • Laboratory connectivity • Mobile facility-level entry	• Human resource requirements • Hardware limitations • Dependency on coordinators
Myanmar	DHIS2, Social media tools	• Standardized reporting format • Facebook-based notification • Social media screening	• Aggregated data only • Internet connectivity • Political instability
Nigeria	e-TB Manager, GxAlert, DHIS2, Commcare, MATS, etc	• Laboratory connectivity • Some open-source solutions • Partner-specific implementations	• Lack of unified approach • Discontinued systems • Funding instability
Pakistan	DHIS2, DHIS2 Tracker	• Transitioning to case-based data • Innovative specimen transport • Pharmacy engagement	• Hardware requirements • Internet connectivity • Change management
Philippines	ITIS ecosystem	• Online/offline capability • Private sector integration • Laboratory connectivity	• Server performance • UHC transition uncertainty • System sustainability
Tanzania	ETL (DHIS2-based)	• Local developer support • Health system integration • Mobile data collection	• Offline functionality • Internet connectivity • Hardware constraints
Vietnam	VITIMES (2023 version)	• Enhanced TB modules • Offline mode in ACIS • Centralized database	• Complex interface • Limited infrastructure • Technical capacity gaps

Digital TB surveillance systems across the ten countries demonstrate significant variation in implementation approaches and technological foundations, reflecting local health system contexts, resource availability, and partnership arrangements.

India’s Nikshay ecosystem (including the Aushadhi and Setu modules) represents the most comprehensive approach, integrating supply chain management, benefit transfers, and digital adherence technologies with patient-centric direct benefit transfers. The Philippines’ sophisticated ecosystem (ITIS (Integrated TB Information System), ITIS Lite, DataToCare, eTBMAC (electronic TB Manager Advisory Committee)) centers on ITIS with specialized components for private sector engagement and laboratory connectivity, though currently transitioning toward Universal Health Care integration.

Kenya’s TIBU (Swahili word meaning “treatment/cure) system (TIBU, TIBU LIMS (Laboratory Information Management System), TIBU Lite), operational since 2012 with one million registered cases, prioritizes data quality and unified reporting across facilities despite requiring significant human resources. Tanzania’s DHIS2 (District Health Information Software 2)-based system benefits from local university development enhancing sustainability, with supplementary USSD-based screening tools demonstrating strong Ministry of Health integration.

Indonesia transitioned from SITT to the real-time SITB (Sistem Informasi Tuberkulosis) in January 2020, following a development phase initiated in 2017 with specialized applications including SITK for contact investigation, WIFI TB for specimen transport, SOBAT TB for child TB cases, and SITRUST for private notifications. Vietnam (VITIMES (Vietnam Tuberculosis Information Management System) in 2023) upgraded with ACIS (Active Case-finding Information System) providing offline capabilities, though expanded functionality creates usability challenges for commune-level staff in remote areas.

Bangladesh’s electronic e-TB Manager, Janao app (from a *Bengali word meaning “inform/notify*), TBCP (TB Control Program) software employs three systems with e-TB Manager providing district-level monitoring limited to lab technicians, Janao app with limited uptake among private providers in urban areas, and TBCP software functioning within Bangladesh Rural Advancement Committee (BRAC) centers without national integration. Myanmar (DHIS2, Facebook Messenger tools) utilizes DHIS2 for aggregated reporting with innovative Facebook Messenger-based notifications, though systems remain predominantly aggregated rather than case-based.

Pakistan (DHIS2, DHIS2 Tracker) transitions from aggregated to case-based reporting with innovative ride-sharing specimen transport and pharmacy-based case detection, with nationwide scale-up of DHIS2 Tracker achieved in 2024. Nigeria (e-TB Manager, GxAlert (GeneXpert Alert system for automated laboratory result notification), various partner platforms) exhibits significant fragmentation with discontinued e-TB Manager and multiple partner-specific platforms due to insufficient national coordination and funding constraints.

Public sector participants emphasized government backing and wide coverage but noted bureaucratic processes. Private sector participants highlighted rapid innovation but cited sustainability and integration challenges when donor funding ended.

### TB technology maturity and feature implementation

**Fig. 3:**
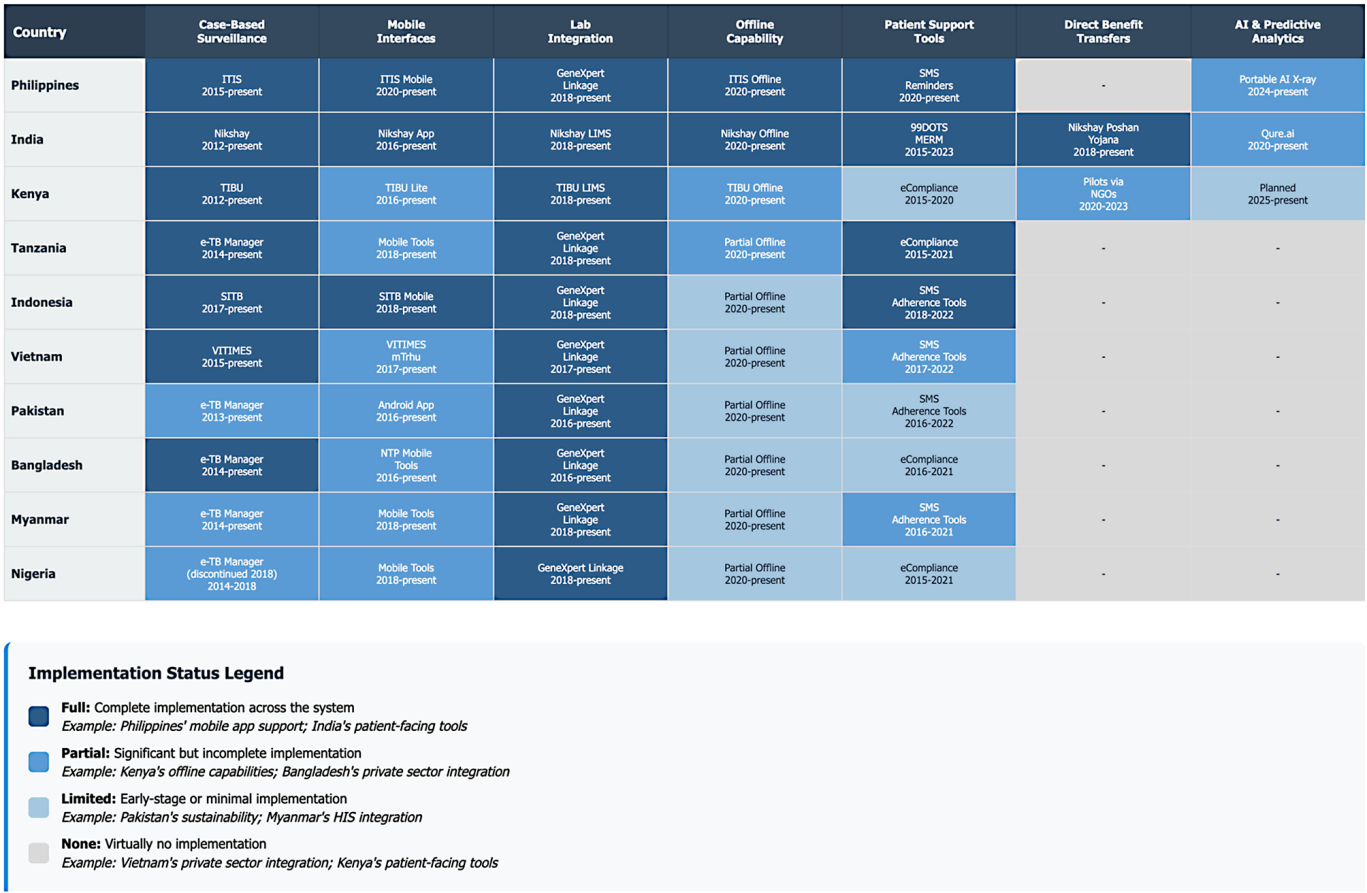
TB technology systems implementation Timeline and status by country

**Fig. 4:**
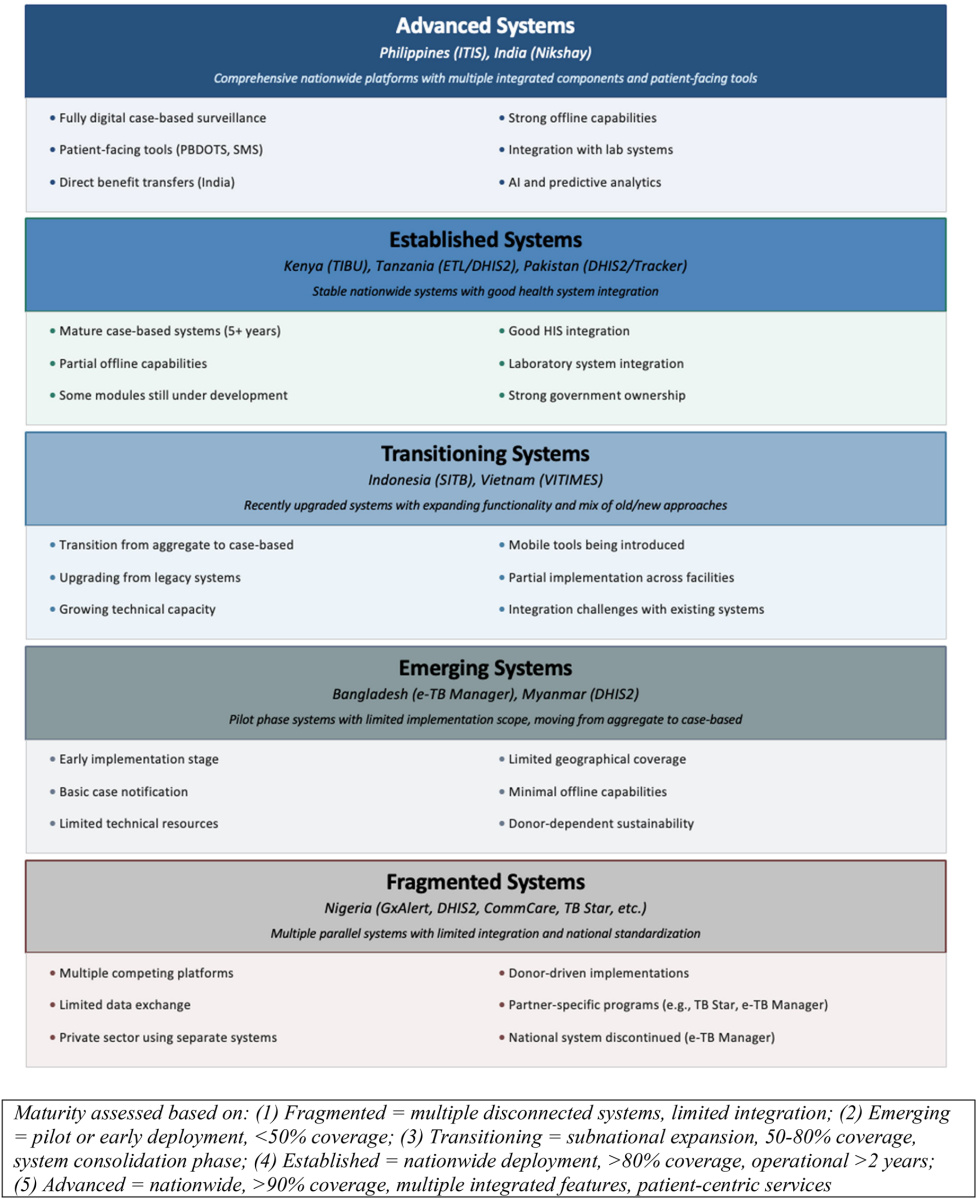
Country TB technologies maturity grouping

Technology implementation across these high-burden countries revealed distinct patterns in platform architecture and functionality (Figs. 3 and 4). Cross-country comparison revealed distinct maturity groupings, with India and the Philippines establishing comprehensive nationwide platforms with patient-centric features and multiple integrated components, while Bangladesh, Myanmar, and Pakistan exemplify emerging systems with pilot implementations gradually moving from aggregated to case-based approaches.

Web-based platforms have revolutionized TB management by centralizing data collection and analysis, significantly enhancing patient tracking and treatment compliance. India’s Nikshay platform demonstrates this evolution, integrating case tracking, treatment monitoring, and adherence management while ensuring accurate reporting and streamlined follow-up [3, 53]. The platform’s progression illustrates the patient-centered trajectory, incorporating patient support integration (2022) and direct benefit transfer systems through the Nikshay Poshan Yojana implemented in 2018 [54]. Similarly, Indonesia’s Sistem Informasi TB (SITB) platform supports real-time data collection and patient management, enabling health authorities to monitor TB cases with greater precision and responsiveness [31, 55]. The latest phase of this evolution has seen AI and computer vision technologies being deployed in 2023–2024, with systems like Qure.ai in India and portable AI X-ray tools in the Philippines showing promise for improving case detection efficiency [56–59]. These comprehensive systems provide a holistic approach by integrating diagnostic and treatment perspectives, enabling more effective and timely care decisions.

Feature implementation analysis revealed that case-based data capability was most widely implemented across countries, while offline functionality remained a persistent challenge even in otherwise advanced systems. Mobile access showed strong implementation in both advanced systems and selective emerging systems that prioritized mobile-first approaches. Private sector integration varied widely, with some emerging systems (Myanmar, Pakistan) demonstrating stronger implementation than more mature systems. Health information system integration was most advanced in established systems (Kenya, Tanzania), highlighting their strength in this specific domain.

### Technology facilitators, barriers, and implementation challenges

Across the ten countries, 29 distinct TB information systems were documented. These ranged from comprehensive national platforms to specialized tools. Table 3 and Appendix B summarize the facilitators (advantages), barriers (disadvantages), and implementation challenges identified by the participants.

**Table 3:** Key facilitators, barriers, and implementation challenges

Facilitators	Barriers	Implementation Challenges
• Real-time data collection • Patient-level tracking • Digital notifications • Standardized reporting • Mobile accessibility • Automated dashboards • Direct benefit transfers • Treatment adherence tracking • Laboratory result integration • Government mandate for digital adoption • Strong leadership and stakeholder support	• Internet dependency • Limited offline functionality • Poor interoperability • Complex user interfaces • Special hardware requirements • Limited private sector adoption • Parallel paper documentation • Data quality concerns • Siloed information systems • Staff attrition and training discontinuity • Personal financial burden on healthcare workers	• Connectivity infrastructure • Hardware access and maintenance • Technical support capacity • Staff training requirements • Sustainable funding • Integration with health systems • Technology literacy gaps • User resistance to change • Geographic implementation disparities • Dedicated data management infrastructure needs

The TB information systems analyzed across the ten high-burden countries revealed distinct patterns of strengths and limitations.

Advantages primarily centered on real-time data accessibility, patient-level tracking capabilities, and standardized reporting frameworks. India’s Nikshay system demonstrated particularly robust patient-centered design, enabling treatment adherence monitoring and direct benefit transfers to patients’ bank accounts. Kenya’s TIBU platform showcased strong data validation mechanisms, with over one million cases registered since 2012, while Philippines’ ITIS offered valuable offline synchronization capabilities critical in island-based geographies.

Disadvantages consistently included connectivity dependencies, with many systems lacking robust offline functionality. Nigeria’s e-TB Manager, despite user appreciation, was discontinued largely due to infrastructural and support limitations. Indonesia’s fragmented ecosystem of multiple specialized systems (SITB, SITK, WIFI TB) created coordination challenges despite technical sophistication. Tanzania’s district-level DHIS2 implementation struggled with last-mile coverage to facilities, while Vietnam’s new VITIMES (2023) introduced complexity barriers for community-level staff (expanded data entry requirements, multiple parallel systems, and increased technical workflows) despite enhanced functionality.

Implementation challenges extended beyond technical limitations to encompass systemic constraints. Myanmar’s political instability directly impacted information system deployment, while Bangladesh’s private practitioner engagement remained limited despite the dedicated Janao notification app. Pakistan adopted ride-sharing for specimen transportation (~800 samples daily), though volunteer rider reliance raised sustainability concerns. The generational divide in technology comfort appeared particularly pronounced in Vietnam and Kenya, necessitating tailored training approaches. These cross-country comparisons highlight how local contexts fundamentally shape implementation outcomes beyond the technologies themselves.

Analysis of implementation facilitators, barriers, and challenges revealed patterns corresponding to the evolutionary stages illustrated in Fig. 1. Systems in early developmental phases (2006–2014) encountered fundamental barriers related to infrastructure limitations and paper-to-digital transition challenges. As technologies advanced to case-based systems (2014–2018), implementation barriers shifted toward user adoption, training needs, and data quality concerns. Most recently, systems incorporating integration capabilities and patient-centered approaches (2018–2025) face more complex challenges related to interoperability with existing health information ecosystems and sustainability of advanced features. Internet dependency emerged as a persistent challenge across all evolutionary phases, though systems with offline capabilities demonstrated greater resilience to this barrier. Notably, while feature implementation has generally progressed chronologically, the uneven implementation across countries suggests that contextual factors significantly moderate technology adoption regardless of development stage.

### CFIR domain analysis

We identified five themes and 19 sub-themes categorized along the five domains of CFIR. The themes are: 1) Characteristics of an effective TB information system, 2) Contextual factors in implementing the technology, 3) Challenges and opportunities within the NTPs, 4) Influence of personal attributes on technology, and 5) Implementation challenges and strategies. Table 4 and Appendix C summarizes these themes, subthemes along with representative excerpts. The codes that formulate each theme are summarized and categorized under their respective domains.

**Table 4:** Themes, sub-themes and implementation factors from CFIR analysis

Sub-themes (CFIR Construct)	Key Implementation Factors	Representative Quotes	Country/System Examples
**I. CFIR Innovation Domain** Characteristics of an effective TB information system			
International collaboration as evidence base (*Evidence-Base*)	• Systems adapted from successful international models show better acceptance • Piloting approach enables refinement before scale-up	*“This is a similar model as of Nikshay. So we got support from Gates Foundation and they supported us with the concept paper and the details of how the call center and how Nikshay is working in India.”*	**Pakistan (PPM Hub):** Adapted from India’s Nikshay **Indonesia (SITB):** Influenced by global partners
Enhanced data quality for patient-centric care (*Relative Advantage*)	• Patient-level tracking provides substantial advantage over aggregate data • Improved data quality enhances decision-making	*“India introduced the case-based surveillance system called Nikshay and that was a major change in terms of the program management.”*	**India (Nikshay):** Shift from aggregate to case-based **Kenya (TIBU):** Patient-level data capture
Real-time reporting for efficient tracking (*Relative Advantage*)	• Immediate data availability eliminates reporting delays • Automated analysis reduces workload for staff	*“I think other benefits that ITIS has provided is the timeliness of the report … the central office already has real time data from the facility.”*	**Philippines (ITIS):** Real-time central monitoring **Indonesia (SITB):** Real-time information system
Integration capabilities with other apps (*Adaptability*)	• Linkage with benefit transfer systems increases perceived value • Compatibility with existing health information systems enhances utility • Multiple parallel systems create need for unified platform	*“The most important thing was the patient support system which was possible only with a digital system like the case-based system of Nikshay.”* *“My suggestion would be to synergise and find a way to put everything on one umbrella … for National reporting … and fit into the main server of the National electronic platform”*	**India (Nikshay):** Integration with benefit transfers **Philippines (ITIS):** Hub for multiple applications **Nigeria (Multiple):** Multiple systems need integration
Technical limitations requiring improvement (*Design*)	• Connectivity requirements limit rural implementation • Performance limitations during peak reporting periods • Offline synchronization capabilities critical for field use	*“Now we are collecting the data but the data is too big. So especially during reporting period we are experiencing some server slow down in the system.”*	**Philippines (ITIS):** Server performance issues **India (Nikshay):** Internet dependency challenges
**II. CFIR Outer Setting** External factors influencing implementation			
National infrastructure context impacts feasibility *(External System & Resources*)	• National electricity grid reliability determines system accessibility • Regional connectivity infrastructure creates urban-rural implementation disparities	*“For those facilities which are in the urban areas where Internet and electricity is reliable, they use ETL. But [rural facilities] have paper tools.”* “*Due to the current situation of the frequent Internet black holes*”	**Tanzania**: Urban facilities connected; rural facilities paper-based **Myanmar:** National connectivity disruptions **Indonesia:** Urban-rural infrastructure disparities
External policies and mandates drive adoption *(Policies & Incentives)*	• Mandatory notification policies drive digital adoption • Government directives requiring system use enhance compliance • National digital health strategies mandate electronic systems	*“There is a government passed law like every TB case should be notified.”* “*In line with the mandate for us to move to the modern technology … to transform from paper based to electronic based … the Ministry of Health provision and mandate*”	**Bangladesh:** Legal notification requirements **Philippines:** Memo mandating ITIS use **Nigeria** (Kano State): Government-mandated digital transition
Donor funding dependency creates implementation vulnerabilities *(External Policies & Incentives)*	• External funding creates sustainability challenges • Donor withdrawal disrupts system continuity • Limited cross-sector collaboration reduces system impact	*“There’s a sizable proportion of staff that are donor funded … if donor funding stops then it may hamper how our service delivery is done.”* *“One year since last year, it has not been working because the grant phased out and the NTLP doesn’t have more … resource to keep it working”*	**Kenya:** Donor dependency risks **Tanzania:** System discontinued after grant ended **Nigeria:** e-TB Manager discontinued after funding ended
**III. CFIR Inner Setting** Organizational characteristics within the NTPs			
NTP organizational strengths support implementation *(Structural Characteristics*)	• Clear role definition enhances implementation • Technical capacity supports system adoption • Staff commitment drives success	*“Our TB program is well structured, very well established and based on that it has a very good technical capacity.”*	**Kenya:** Well-defined roles and responsibilities **Pakistan:** Technical leadership commitment
Organizational infrastructure and capacity (*Available Resources*)	• Hardware availability affect system performance • Server capacity determines scalability • Staff training capacity and continuity • Personal financial burden on staff for connectivity costs	*“These apps need internet in some parts of the country there is very limited internet access.”* *“if you have a staff … and then he has been working on this for some time and … it is time for retirement or posting then you begin to scrap … The issue of training has not been consistent”* ”*It was really tough because a lot of people were using their personal money to maybe add and top up data.”*	**Bangladesh:** Staff connectivity costs **Vietnam:** Outdated organizational hardware **Indonesia:** Server capacity challenges **Nigeria**: Staff attrition and training gaps
Previous system limitations driving change (*Readiness for Implementation - Tension for Change*)	• Aggregated data limitations created demand for case-based systems • Reporting delays motivated real-time solutions • Analysis difficulties drove dashboard development	*“The challenge with DHIS2 tracker was it was only providing aggregated data … we will not be able to follow up with those cases.”*	**Pakistan (DHIS2):** Limitations of aggregate data **India:** Quarterly reporting delays
Internal policies requiring adaptation (*Compatibility*)	• Digital transition requires internal policy updates • Validation processes need adaptation for new systems • Workflow procedures must align with digital systems	“When we shift from paper-based to digital system … we also need to revise our systems and policies.”	**Pakistan**: Internal policy updates needed for digital transition
Incentive structures for adoption *(Implementation Climate)*	• Financial incentives enhance system utilization • Recognition programs motivate performance	*”1000 rupees for each patient. 500 for notification and 500 on treatment completion.”* ”*The incentives are good because it can motivate reporting to ensure timely reporting … incentives support many other things that can make the work happen.*”	**India (Nikshay):** Financial notification incentives **Philippines (ITIS):** Performance recognition awards **Nigeria**: Incentives for reporting
**IV. CFIR Individual Domain** Influence of personal attributes on technology			
Individual comfort with technology varies (*Knowledge and Beliefs*)	• Technology familiarity varies across staff generations • General digital literacy affects system-specific adoption • Implementation champions emerge as critical success factors	*“People are very well-versed nowadays, not just the younger generations but even the older generation in India.”*	**India:** Cross-generational technology comfort **Vietnam:** Age-related adoption barriers
**V. CFIR Implementation Process** Strategies and approaches for system deployment			
Attitudes toward change affecting adoption (*Engaging*)	• Resistance often stems from workflow disruption • Parallel paper-digital systems create duplication burden • User perception of value determines adoption	*“It is not the difficulty in using Nikshay, it is the reluctance sometimes.”*	**India (Nikshay):** User reluctance despite usability **Pakistan:** Resistance to digital transition
Structured implementation strategies (*Planning & Executing*)	• Requirements analysis improves system-context fit • Stakeholder engagement enhances ownership • User feedback mechanisms enable refinement • Extended support hours address practical barriers	*“For a new technology, first we are doing requirements analysis and then gathering all stakeholders involved.”*	**Philippines (ITIS):** Structured implementation **Bangladesh:** Extended data entry support **Pakistan:** Continuous feedback loops
Training and transition support (*Executing*)	• Online resources enhance accessibility • Database conversion support facilitates transition • Technical guidance materials improve user competence	”*We have the video tutorial for using SITB … uploaded in YouTube as well as the technical guideline stored in the online drive*.”	**Indonesia** (SITB): Online training resources **Philippines**: Database conversion support

In the *Innovation* Domain, effective TB information systems demonstrated international collaboration as evidence base, with India’s Nikshay influencing development in other countries like Pakistan’s PPM Hub. Enhanced data quality through patient-level tracking represented a significant relative advantage over aggregate systems. Real-time reporting capabilities, as seen in Philippines’ ITIS, enabled immediate central office access to facility data. Systems with integration capabilities showed greater utility, though technological limitations persisted, particularly regarding connectivity requirements and performance issues.

The *Outer Setting* significantly influenced implementation through local conditions and policy frameworks. Political stability and infrastructure created enabling or constraining environments. Mandatory notification policies (Bangladesh) and directed use of specific systems (Philippines’ ITIS memo) provided regulatory foundations for adoption, though implementation success varied with enforcement capacity.

Within the *Inner Setting* domain, we identified both challenges and opportunities. Organizational strengths of well-established TB programs with clearly defined roles provided implementation foundations, while donor dependencies created sustainability risks (Nigeria’s discontinued e-TB Manager). Infrastructure limitations—particularly internet connectivity and server capacity—consistently hindered full system utilization across all countries. Previous system limitations created tension for change, with incentive systems and training resources serving as enablers.

The *Individual* Domain theme focused on personal attributes influencing technology adoption. Increasing comfort with technology simplified system use, though generational patterns emerged across countries. While India reported widespread technology comfort across age groups, Vietnam and Kenya noted significant digital literacy gaps between younger and older staff.

The *Implementation Process* theme revealed how attitudes toward change affected adoption, with resistance most pronounced where systems required substantial workflow modifications. Efficient implementation processes facilitated by strategies like Philippines’ bi-annual user conferences, Pakistan’s continuous feedback mechanisms, and Bangladesh’s extended support hours addressed practical implementation barriers.

**Fig. 5:**
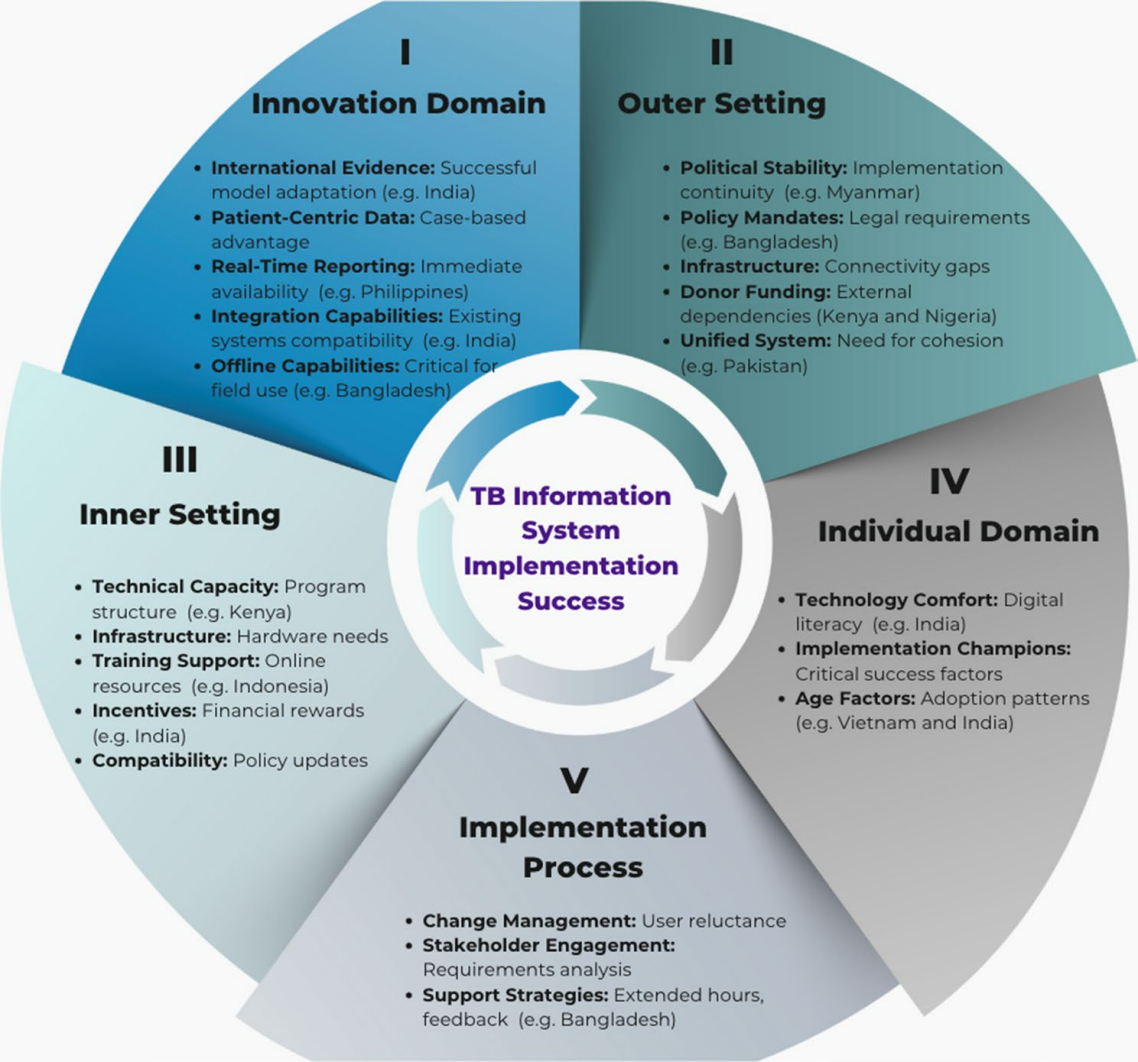
Factors influencing TB technology implementation success

The CFIR analysis framework, visualized in Fig. 5, illuminates how implementation challenges interact across multiple domains to influence overall success. Barriers identified in our analysis show varying impact patterns depending on their domain position and relationship strength. The relationship between technological limitations (Innovation Domain) and implementation processes demonstrates particularly strong influence, explaining why offline functionality remains challenging despite its recognized importance. Implementation facilitators show similar cross-domain interaction effects; for instance, strong program structures (Inner Setting) amplify the positive impact of user feedback mechanisms (Implementation Process). The relative strength of connections between domains helps explain why countries with similar technological sophistication achieved different implementation outcomes. This cross-domain interaction pattern supports our core finding that implementation success depends more on alignment across all CFIR domains than on technological sophistication alone—a pattern evident when comparing countries like India and Nigeria, where similar technologies yielded dramatically different implementation results due to varying contextual alignment.

These findings across the 43 CFIR constructs within 5 domains highlight that successful TB information system implementation requires multidimensional alignment between innovation characteristics, contextual factors, organizational capacity, individual attributes, and implementation processes.

## Discussion

The comparative analysis of TB information systems across high-burden countries reveals several significant patterns with implications for future implementation efforts. Implementation status varies considerably, with only three countries (India, Kenya, and Pakistan) achieving widespread implementation. Three countries demonstrate moderate implementation (Philippines, Indonesia, Tanzania), while four (Bangladesh, Vietnam, Nigeria, and Myanmar) show limited implementation despite years of development in some cases. Implementation maturity was assessed using a 5-level framework derived from participants descriptions: (1) Fragmented systems with multiple disconnected platforms and limited integration (e.g., Nigeria); (2) Emerging systems in pilot or early deployment with <50% coverage (e.g., Bangladesh, Myanmar); (3) Transitioning systems undergoing subnational expansion with 50–80% coverage and system consolidation (e.g., Indonesia, Vietnam); (4) Established systems with nationwide deployment, >80% coverage, and operational > 2 years (e.g., Kenya, Tanzania, Pakistan); and (5) Advanced systems with nationwide reach, >90% coverage, multiple integrated components, and patient-centric services (e.g., Philippines, India). This framework emerged inductively from participants’ descriptions and aligns with established implementation science constructs regarding reach and penetration [60], sustainability [61], and system complexity [62].

### Health systems and policy implications

The variations in implementation success across countries highlight critical health systems factors that influence digital health deployment in LMIC settings. Countries with stronger health system governance structures—evidenced by clear role delineation, regulatory frameworks, and multi-stakeholder coordination—demonstrate more successful implementation outcomes. This finding has important implications for health policymakers considering digital health investments, as it suggests that health systems strengthening may be a prerequisite for sustainable technology deployment.

### Evolution and current state of TB information systems

The transition to digital systems across all studied countries align with global strategies for TB elimination that emphasize the importance of robust information systems [1]. Most countries have developed or implemented case-based TB information systems, moving beyond the aggregated reporting systems previously common in TB programs. Our findings reveal that evolution pace varied significantly based on governance structures rather than resource availability alone, highlighting that LMICs do not face uniform adoption barriers [6, 63].

Digital health solutions can bridge gaps in TB care by improving case notification, adherence monitoring, and data analysis [14, 64]. Our findings confirm this potential while also highlighting implementation challenges. For instance, Bangladesh’s Janao system and Pakistan’s PPM Hub demonstrate how mobile technologies can facilitate TB notification from private providers, addressing a critical gap in TB surveillance.

The latest evolution phase has seen AI and computer vision technologies being deployed starting in 2023–2024, with systems like Qure.ai in India and portable AI X-ray tools in the Philippines showing promise for improving case detection efficiency [56, 57, 59]. Emerging technologies present additional opportunities for TB program optimization, including automated communication systems for standardizing referral processes and location-based services for strategic resource placement. Geographic information systems integrated with provider networks could enhance NTP planning capabilities and enable community-driven accountability mechanisms, though implementation of such innovations remains in early stages.

These information systems provide a holistic approach by integrating and synchronizing data from both diagnostic and treatment perspectives, enabling more effective and timely care decisions. Beyond surveillance and case management, the most advanced systems have begun incorporating direct patient interaction capabilities, marking a significant shift from provider-focused to patient-inclusive digital health approaches [65].

The integration of patient support mechanisms with information systems represents a transformative development, particularly evident in systems with widespread implementation. This integration fundamentally reorients TB care toward more comprehensive patient-centered approaches, enabling direct benefit transfers and enhancing treatment adherence monitoring, for patient-centered eHealth systems that support the entire TB care cascade [7].

While these advances demonstrate significant potential, our findings align with established literature regarding persistent implementation challenges. However, the adoption of digital systems is not without challenges. Firstly, economic barriers complicate the implementation of digital TB surveillance systems in resource-limited settings [1, 66]. The initial investment costs, ongoing maintenance requirements, and need for technical support create sustainability challenges for many high-burden countries [67]. Additionally, reliance on stable internet connectivity can be problematic in low-resource or rural areas with inadequate infrastructure, disrupting the functionality of web-based platforms and mobile apps, affecting data access and patient monitoring [38].

Additionally, digital literacy remains a significant barrier, as healthcare workers and patients in underserved areas may lack the skills to effectively use these technologies, limiting their impact [38]. Resistance to change, inadequate policies and governance structures, insufficient financial resources, and concerns about data privacy and security further impede successful deployment [38, 68, 69]. Without clear guidelines and effective management, implementation may be inconsistent and poorly coordinated [38].

### Implementation factors through CFIR lens

Our analysis of TB information systems through the CFIR framework revealed that implementation success depends less on technological sophistication than on alignment across all domains. Systems achieving widespread implementation (India, Philippines, Kenya) demonstrated stronger performance across innovation characteristics (technological adaptability, evidence base), outer setting factors (policy mandates, external partnerships), inner setting readiness (organizational capacity, resource availability), individual factors (digital literacy, user engagement), and implementation process (planning quality, stakeholder involvement).

Conversely, countries with fragmented implementations faced challenges spanning multiple domains simultaneously. For example, Pakistan’s political instability (outer setting) combined with infrastructure limitations (inner setting) constrained implementation despite technological innovation. This finding underscores that digital health implementation requires holistic health systems strengthening rather than isolated technological interventions, as connectivity constraints and workflow integration challenges persist across context [13, 70–75]. Successful implementations demonstrated platform flexibility through dual online/offline capabilities and mobile interfaces that overcome infrastructure constraints [2, 65], though sustainability requires policy mandates paired with consistent funding beyond initial technology investments [76].

### Implications for health policymakers and TB programs

Our findings suggest several critical implications for health policymakers and NTP managers. First, policymakers must consider the total cost of ownership for digital systems, including not only initial technology costs but also ongoing infrastructure, capacity building, and maintenance requirements, beyond initial technology costs. Sustainable financing models are essential, as evidenced by implementation challenges in donor-dependent contexts.

Our CFIR analysis revealed that implementation success was associated with multi-stakeholder coordination mechanisms. Countries demonstrating more mature implementations (India, Philippines, Kenya, Tanzania) described formal coordination structures that aligned national TB programs, ministries of health, technical partners, and donors around common goals. For example, the Philippines’ ITIS ecosystem involves coordination between the Department of Health, implementing partners, and private sector providers through structured governance mechanisms. System integrations with direct benefit transfer programs required coordination across multiple government ministries. In contrast, countries with more fragmented implementations described various barriers, like challenges establishing consistent coordination across stakeholders, and discontinuation reflecting sustainability challenges when donor funding ended despite having coordination structures. These findings suggest that while collaborative governance is not sufficient for implementation success, the presence of formal coordination structures was a consistent feature among systems achieving broader geographic coverage and sustained operation.

## Limitations and future research

This study has several limitations. Our reliance on key informant interviews with TB program implementerss may have introduced bias toward official perspectives, and we did not conduct technical evaluations of system functionality. Future research should incorporate end-user experiences, particularly from frontline healthcare workers and patients, assess system performance objectively, and examine impacts on TB program metrics including case notification and treatment outcomes.

Our study achieved a 50% response rate (15 of 30 invited participants), which may introduce selection bias. Non-respondents may have differed systematically from participants, potentially biasing findings toward individuals with more positive implementation experiences, greater availability for interviews, or stronger connections to international TB networks. However, several factors suggest our findings capture a meaningful range of implementation experiences. First, we achieved representation across all 10 target countries, ensuring geographic diversity. Second, our sample included both public sector participants (*n* = 6, 40%) and private sector participants (*n* = 9, 60%), reflecting the diversity of implementation contexts. Third, participants represented diverse roles including program management, monitoring and evaluation, and technical implementation, providing multiple perspectives on implementation challenges. Fourth, participants from countries with known implementation challenges (Myanmar, Nigeria, Pakistan) discussed substantial barriers openly, suggesting our sample was not limited to success stories. Finally, our purposive sampling strategy prioritized information-rich cases—individuals with direct implementation experience—rather than representative sampling, which is appropriate for qualitative research aimed at understanding implementation phenomena rather than estimating population parameters. Nevertheless, findings should be interpreted recognizing that non-response may have influenced which implementation challenges and facilitators were most prominently represented in our data

## Conclusion

Digital TB information systems in high-burden countries have evolved significantly, yet our multi-country analysis reveals that multi-factor implementation readiness, rather than technological sophistication alone, determine success. Twenty-nine distinct systems across 10 countries, demonstrated that health systems factors, including infrastructure, governance, and capacity critically shape sustainable technology deployment.

Successful implementation requires coordinated health systems strengthening. Countries with better outcomes established collaborative governance frameworks aligning national TB programs, ministries, technical partners, and donors around common goals.

Key policy implications include: implementation readiness assessment must precede technology selection; countries should prioritize contextual adaptation, technical leadership development, and user-centered design; and sustainable financing mechanisms are essential.

Critical questions remain: how can digital health investments support health equity and patient-centered care rather than purely efficiency-driven outcomes? Future research should develop practical implementation frameworks that translate these findings into actionable guidance enabling evidence-based digital health investments that strengthen TB elimination efforts and health systems resilience.

### Appendix A

**Table 5:** Domains and constructs of CFIR

I. Innovation Domain	
A. Innovation Source	The group that developed and/or visibly sponsored use of the innovation is reputable, credible, and/or trustable.
B. Innovation Evidence-Base	The innovation has robust evidence supporting its effectiveness.
C. Innovation Relative Advantage	The innovation is better than other available innovations or current practice.
D. Innovation Adaptability	The innovation can be modified, tailored, or refined to fit local context or needs.
E. Innovation Trialability	The innovation can be tested or piloted on a small scale and undone.
F. Innovation Complexity	The innovation is complicated, which may be reflected by its scope and/or the nature and number of connections and steps.
G. Innovation Design	The innovation is well designed and packaged, including how it is assembled, bundled, and presented.
H. Innovation Cost	
**II. Outer Setting Domain**	
A. Critical Incidents	Large-scale and/or unanticipated events disrupt implementation and/or delivery of the innovation.
B. Local Attitudes	Sociocultural values (e.g., shared responsibility in helping recipients) and beliefs (e.g., convictions about the worthiness of recipients) encourage the Outer Setting to support implementation and/or delivery of the innovation.
C. Local Conditions	Economic, environmental, political, and/or technological conditions enable the Outer Setting to support implementation and/or delivery of the innovation.
D. Partnerships & Connections	The Inner Setting is networked with external entities, including referral networks, academic affiliations, and professional organization networks.
E. Policies & Laws	Legislation, regulations, professional group guidelines and recommendations, or accreditation standards support implementation and/or delivery of the innovation.
F. Financing	Funding from external entities (e.g., grants, reimbursement) is available to implement and/or deliver the innovation.
G. External Pressure	External pressures drive implementation and/or delivery of the innovation. Note: Use this construct to capture themes related to External Pressures that are not included in the subconstructs below.
—1. Societal Pressure	Mass media campaigns, advocacy groups, or social movements or protests drive implementation and/or delivery of the innovation.
—2. Market Pressure	Competing with and/or imitating peer entities drives implementation and/or delivery of the innovation.
—3. Performance-Measurement Pressure	Quality or benchmarking metrics or established service goals drive implementation and/or delivery of the innovation.
**III. Inner Setting Domain**	
A. Structural Characteristics	Infrastructure components support functional performance of the Inner Setting. Note: Use this construct to capture themes related to Structural Characteristics that are not included in the subconstructs below.
—1. Physical Infrastructure	Layout and configuration of space and other tangible material features support functional performance of the Inner Setting.
—2. Information Technology Infrastructure	Technological systems for tele-communication, electronic documentation, and data storage, management, reporting, and analysis support functional performance of the Inner Setting.
—3. Work Infrastructure	Organization of tasks and responsibilities within and between individuals and teams, and general staffing levels, support functional performance of the Inner Setting.
B. Relational Connections	There are high quality formal and informal relationships, networks, and teams within and across Inner Setting boundaries (e.g., structural, professional).
C. Communications	There are high quality formal and informal information sharing practices within and across Inner Setting boundaries (e.g., structural, professional).
D. Culture	There are shared values, beliefs, and norms across the Inner Setting. Note: Use this construct to capture themes related to Culture that are not included in the subconstructs below.
—1. Human Equality-Centeredness	There are shared values, beliefs, and norms about the inherent equal worth and value of all human beings.
—2. Recipient-Centeredness	There are shared values, beliefs, and norms around caring, supporting, and addressing the needs and welfare of recipients.
—3. Deliverer-Centeredness	There are shared values, beliefs, and norms around caring, supporting, and addressing the needs and welfare of deliverers.
—4. Learning-Centeredness	There are shared values, beliefs, and norms around psychological safety, continual improvement, and using data to inform practice.
Note:	Constructs E – K are specific to the implementation and/or delivery of the innovation.
E. Tension for Change	The current situation is intolerable and needs to change.
F. Compatibility	The innovation fits with workflows, systems, and processes.
G. Relative Priority	Implementing and delivering the innovation is important compared to other initiatives.
H. Incentive Systems	Tangible and/or intangible incentives and rewards and/or disincentives and punishments support implementation and delivery of the innovation.
I. Mission Alignment	Implementing and delivering the innovation is in line with the overarching commitment, purpose, or goals in the Inner Setting.
J. Available Resources	Resources are available to implement and deliver the innovation. Note: Use this construct to capture themes related to Available Resources that are not included in the subconstructs below.
−1. Funding	Funding is available to implement and deliver the innovation.
−2. Space	Physical space is available to implement and deliver the innovation.
−3. Materials & Equipment	Supplies are available to implement and deliver the innovation.
K. Access to Knowledge & Information	Guidance and/or training is accessible to implement and deliver the innovation.
**IV. Individuals Domain**	
A. High-Level Leaders	Individuals with a high level of authority, including key decision-makers, executive leaders, or directors.
B. Mid-level Leaders	Individuals with a moderate level of authority, including leaders supervised by a high-level leader and who supervise others.
C. Opinion Leaders	Individuals with informal influence on the attitudes and behaviors of others.
D. Implementation Facilitators	Individuals with subject matter expertise who assist, coach, or support implementation.
E. Implementation Leads	Individuals who lead efforts to implement the innovation.
F. Implementation Team Members	Individuals who collaborate with and support the Implementation Leads to implement the innovation, ideally including Innovation Deliverers and Recipients.
G. Other Implementation Support	Individuals who support the Implementation Leads and/or Implementation Team Members to implement the innovation.
H. Innovation Deliverers	Individuals who are directly or indirectly delivering the innovation.
I. Innovation Recipients	Individuals who are directly or indirectly receiving the innovation.
**V. Implementation Process Domain**	
A. Teaming	Join together, intentionally coordinating and collaborating on interdependent tasks, to implement the innovation.
B. Assessing Needs	Collect information about priorities, preferences, and needs of people. Note: Use this construct to capture themes related to Assessing Needs that are not included in the subconstructs below.
—1. Innovation Deliverers	Collect information about the priorities, preferences, and needs of deliverers to guide implementation and delivery of the innovation.
—2. Innovation Recipients	Collect information about the priorities, preferences, and needs of recipients to guide implementation and delivery of the innovation.
C. Assessing Context	Collect information to identify and appraise barriers and facilitators to implementation and delivery of the innovation.
D. Planning	Identify roles and responsibilities, outline specific steps and milestones, and define goals and measures for implementation success in advance.
E. Tailoring Strategies	Choose and operationalize implementation strategies to address barriers, leverage facilitators, and fit context.
F. Engaging	Attract and encourage participation in implementation and/or the innovation. Note: Use this construct to capture themes related to Engaging that are not included in the subconstructs below.
—1. Innovation Deliverers	Attract and encourage deliverers to serve on the implementation team and/or to deliver the innovation.
—2. Innovation Recipients	Attract and encourage recipients to serve on the implementation team and/or participate in the innovation.
G. Doing	Implement in small steps, tests, or cycles of change to trial and cumulatively optimize delivery of the innovation.
H. Reflecting & Evaluating	Collect and discuss quantitative and qualitative information about the success of implementation. Note: Use this construct to capture themes related to Reflecting & Evaluating that are not included in the subconstructs below.
—1. Implementation	Collect and discuss quantitative and qualitive information about the success of implementation.
—2. Innovation	Collect and discuss quantitative and qualitative information about the success of the innovation.
I. Adapting	Modify the innovation and/or the Inner Setting for optimal fit and integration into work processes.

### Appendix B

**Table:** 

Country	Technology	Advantages	Disadvantages	Implementation Challenges	Representative Quotes
Bangladesh	e-TB Manager	• Digital patient database • Mandatory nationwide system • District monitoring dashboard • Universal platform	• Lab technician access only • Requires computers • No offline capability • Private facilities excluded	• Limited operational hours • Technical staff needs • Training requirements	*“e-TB manager is totally handled by the lab technicians who are giving all the information they have in their register regarding a patient.”*
	Janao app	• Mobile notification app • Easy case reporting • NTP dashboard visibility • Tracks referral sources	• Limited adoption • No offline functionality • Time burden for doctors • Urban area focus	• Internet dependency • Time-consuming data entry • Insufficient sensitization • Funding constraints	*“Janao is a Bengali word. Janao means to let you know or to inform … whenever the practitioners get the case they should notify it to the national system.”*
	TBCP software	• Electronic medical records • Demographic data capture • Diagnostic algorithm support • Test report storage	• BRAC centers only • Poor e-TB Manager integration • Duplicate record risks • No treatment registration	• Pre-implementation training • Diagnostic facility focus • No offline capability • TPT module lacking	*“TBCP software is like a small ERP system. Whenever the presumptives coming to diagnostic facilities, their information are taking in the software like their demographic information and symptoms.”*
India	Nikshay	• Case-based surveillance • Real-time monitoring • Patient-centric approach • Direct benefit transfers • Analytics dashboard	• Internet dependency • Data loss issues • Dual reporting burden • Complex for some users	• Nationwide scaling • Training needs • Tech literacy gaps • Data consistency challenges	*“The most important thing is the patient support system which was possible only with such a digital system like the case-based system of Nikshay.”*
	Nikshay Aushadhi	• Drug supply chain management • Manufacturer-to-patient tracking • Program management support • Logistics monitoring	• Partial Nikshay integration • Unreliable updates • Stock disruptions persist	• Separate development • Integration challenges • Maintenance requirements	*“Nikshay Aushadhi is another application which takes care of the whole drugs and logistics systems at all levels right from the manufacturers to the supplies.”*
	Nikshay Setu	• Bank account validation • Self-screening tool • Patient counseling • Training resources	• Limited training support • Internet dependency • Smartphone requirement	• Patient training needs • Tech literacy barriers • Internet connectivity issues	*“The most important thing was the patient support system … possible … only with such a digital system … if you want to give conditional … direct benefit transfer to 2.5 million patients … it’s not possible with the existing traditional system … cheque or physical cash; there are chances of leakages … even if we have the intentions correct … With the Nikshay there is nothing like that and we can really move ahead with the … direct benefit transfer … the bank accounts details are seeded and we can just move ahead.”*
	99DOTS/MERM	• Adherence monitoring • “Proxy pill in hand” function • Nikshay integration • Automatic recording	• Special hardware needed • Patient compliance dependent • Hardware distribution issues	• Patient training • Adoption challenges • System integration	*“The patient box is there and every time the patient opens the box then the reminder will be automatically gets into the application and it gets reflected in Nikshay.”*
	Epicenter	• First digital TB system (1998) • Standardized reporting format • Global indicator monitoring • Digital infrastructure foundation	• Aggregated data only • Quarterly reporting delays • Limited analytical capability • District-level focus only	• DOS-based initially • Local installation needed • Email-based user access	*“Till 2012, it was aggregated recording, reporting of case finding reports, smear conversion reports, treatment outcome reports and program management reports. These were the major hallmarks of the surveillance system … the reporting was mainly the quarterly reporting by the districts and the states … So you can say there was a delay in overall recording, reporting, and action which completes the loop of the whole program implementation cycle.”*
Indonesia	SITB	• Real-time data collection • Patient tracking system • Centralized TB database	• Complex system design • Internet/server dependent • Performance limitations	• Server capacity issues • Extensive training needs • Connectivity challenges	*“So from the 2014, up until 2020, we use those kinds of information systems that is not really real times … But, today start from 2022, we use the newest information system, we call it SITB, and it’s supposed to be the real-time TB information system for the country itself.”*
	SITK	• Contact tracing focused • SITB data integration • Contact investigation tracking	• Limited government access • Basic functionality only	• Main system integration • Data transfer issues	*“SITK, the supporting information system for contact investigation … they send data from the SITK to SITB.”*
	WIFI TB	• User-friendly interface • Mobile platform • Mandatory notification system • Easy case reporting	• Partial SITB integration • Limited functionality	• Older user adoption issues • Integration challenges	*“It is simple, and mobile, and is not it doesn’t have the buffering process like the SITB one.”*
	SOBAT TB	• Simple user interface • Public accessibility • Self-screening tool	• Limited SITB integration • Restricted data sharing	• Main system integration • User awareness barriers	*“For the screening, SOBAT TB, I think it is easy to use … it is easy to use and users friendly.”*
	SITRUST	• Specimen collection management • Transport logistics system • Sample tracking capability	• Partial SITB integration • Incomplete NTP handover	• Ownership issues • Transition challenges	*“We can just put our order in those applications and courier will come into those facilities and then pick up this specimen and transfer it to the expert site.”*
	ACIS	• Offline capability • Mobile-friendly • Screening and case management • Self-screening tools	• Limited VITIMES integration • Low adoption rate • Parallel system	• Limited implementation • Training requirements • Integration challenges	*“ACIS, in the past was developed by Clinton Health Access Initiative and they gave it to, they share to NTP to use for the NTP active case filing.”*
Kenya	TIBU	• Case-based data system • Data quality validation • Duplicate identification • Analytics capabilities • 1 M+ cases since 2012	• Human resource intensive • Facility visits required • Double data entry burden • Server costs	• Computer literacy gaps • Infrastructure expenses • Connectivity issues • Technology upgrade costs	*“We have a system called TIBU that we use to report for notification of case-based data. So we have around one million people online listed for TB since 2012 when we started the system. So that has been very critical in making sure that we are having unified reports.”*
	TIBU LIMS	• Lab results integration • Real-time GeneXpert data • Clinician notifications • TIBU system integration	• Internet dependency • GeneXpert connectivity issues	• Hardware needs • Technical support • Training requirements	*“TIBU LIMS that has got diagnostic information from GeneXperts and this one is for basically the lab and for purposes of getting results from GeneXpert. So that gives notification for the results to the clinician as soon as the results are available.”*
	TIBU Lite	• Facility-level data entry • Mobile-based (Android) • Reduces data duplication • TIBU system integration	• Personal device reliance • Staff turnover disruptions • Android requirement	• Facility hardware needs • Connectivity issues • Staff transition challenges • Training needs	*“TIBU lite is supposed to be facility-based really unlike TIBU where we used to have the sub county coordinators going around to recapture data from the registers as a tour every month. But this one is direct entry into the system.”*
Myanmar	DHIS2	• Standardized reporting • Centralized data access • Region-specific reports • Nationwide platform	• No notification integration • Aggregate data only • Limited patient tracking • Online-only system	• Connectivity challenges • Digital literacy gaps • Resource limitations • Political environment impact	*“The advantage of DHIS2 national reporting system is we all have the standardized format which can be easily accessed by anyone anywhere who can access the internet. So all the national data can be seen consolidatedly in the DHIS2.”*
	Notification System (chatbot, NODIS)	• Facebook Messenger-based • User-friendly interface • Real-time notifications • Minimal data requirements	• Limited TB adoption • Not nationally implemented • Private sector focus • Internet dependent	• Digital literacy barriers • Connectivity constraints • Private sector engagement • Paper system competition	*”[NODIS] is user friendly tools because it was based on Facebook messenger and later it could be more widely used by all the relevant stakeholders. But currently it is not widely used. When the mandatory notification is compulsory, it will be very useful.”*
	Social Media Screening	• Self-screening tools • Popular platform integration • Diagnostic center referrals • Public awareness tool	• Internet dependency • Smartphone requirement • Tech literacy barriers • Limited adoption	• Connectivity issues • Device access • Awareness challenges • Technical maintenance	*“In Myanmar we use the social media platform to make the raising the public awareness and also we have the chatbot in the Facebook Messenger to the people to get a self-diagnosing if they have the suspect of TB and get assisted referral to the diagnostic center.”*
Nigeria	e-TB Manager	• Case-based reporting • User-friendly interface • Offline capability • Care cascade tracking	• Not open source • Limited tech support • Poor interoperability • Special equipment needs	• Discontinued (2018) • Maintenance problems • External support dependency • User incentive gaps	*“e-TB manager … [was] a patient management tool … both the role of reporting your patient but also play the role that it allows for patient management … it allows you to have feasibility to patient management, how patient are managed, you know along the TB cascade.”*
	GxAlert/GxAspect	• Real-time result alerts • Faster turnaround time • Cartridge management • Equipment monitoring • No special hardware	• Internet dependency • Limited data fields • Limited indicators	• Remote connectivity issues • Evolution to GxAspect	*“GxAlert. The advantage is that is able to alert the care provider and the patient itself about their test result … by tomorrow morning I’m getting an alert that one of them has drug resistant TB … it helps in the management of cartridges, cartridge and logistics management for gene experts.”*
	DHIS2	• Open source • Strong interoperability • User-friendly interface • Data visualization • Technical support	• Not patient-focused • Aggregate data only	• Partner use only • HIV program adaptation	*“DHIS2 can link to so many things, so many, yes, so many.”*
	Commcare	• e-TB Manager equivalent • Patient-level data	• Not open source • Limited coverage	• KNCV exclusive • Limited deployment • Special hardware needs	*“Commcare helps works almost like the e-TB manager that can also provide patient level information and management like the e-TB manager.”*
	MATS App	• Effective screening • Care cascade tracking • Community-level use	• Screening-only • No patient management	• IHVN exclusive • Special equipment needed	*“The MAT app which is also … more of a screening tool … helps you in terms of TB cascade.”*
	EWORS/EPCON	• TB hotspot alerts • Simple implementation • No special equipment • Easy deployment	• Alert-only functionality	• Partner-specific tools • NTP adoption of EWORS	*“It’s capacity to able to enhance the country’s ability to increase its case notification.”*
	TB Star	• Commcare equivalent	• Limited adoption • Discontinued support	• SHOPS project tool • Private sector only	*“For TB star … it also has the same capacity like Commcare from the little I know.”*
Pakistan	DHIS2	• Aggregated sector data • Global platform standard • Internet-based access • Analytics tools	• Aggregate data only • Limited case management • Partial surveillance • No treatment tracking	• Internet requirement • Technical support gaps • User capacity issues • Integration challenges	*“The absence of digital tools, they require the healthcare providers to manually record and document the TB related data … which is a very time-consuming process which sometimes often can delay the data collection process or leading to outdated information and hindering the decision-making processes.”*
	DHIS2 Tracker	• Case-based data • Real-time monitoring • Data quality improvement • Individual case tracking	• Training requirements • Change management issues • Internet dependency • Pilot phase limitations	• User resistance • Training needs • Limited connectivity • Hardware requirements	*“We piloted some years back a specimen transportation model. This transportation model or intervention model, it’s basically based on a technology which includes an android-based mobile application which moderates a right management system.”*
	PPM Hub	• Real-time registration • Follow-up system • Adherence monitoring • Contact screening	• Private sector focus • Limited DHIS2 integration • Pilot phase status • Mobile requirements	• DHIS2 integration • Staff training • Mobile technology needs • Capacity building	*“We have developed a PPM Hub through which real-time TB case registrations, their follow-up, their treatment adherence, contact screening, and counselling is ensured.”*
	Specimen Transportation App	• Mobile specimen system • Uber-like model • Real-time tracking • 800+ daily samples	• Smartphone requirement • Internet dependency • Registered rider need	• Volunteer rider recruitment • System maintenance • Connectivity issues	*“This is an online system which provides real time information on the number of samples that are transported and tested … And on average around 800 samples are transported daily from both public and private facilities.”*
Philippines	ITIS	• Online/offline capability • All TB types coverage • Real-time data collection • Automated reporting	• Server performance issues • Large dataset burden • Complex inventory system • Duplicate data entry	• Internet access • Device requirements • Training needs • Technical support	*“The advantage of ITIS is that in terms of case management, it’s very good because everything in the entire TB cascade of care, everything is there, but it’s really TB specific and when you try to connect system to it, there are challenges.”*
	ITIS Lite	• Private sector simplified • One-page notification • Lower entry barrier • Free to implement	• Limited functionality • Reduced dataset • Backend limitations	• Private sector uptake • EMR integration • Training requirements	*“For the private we develop like a subsystem that is called the ITIS Lite since the data sets in ITIS is very large. So for the national TB program to gain participation from the private sector, they designed like a one pager TB notification form.”*
	End TB Suite	• Mobile app collection • Dashboard tools • Screening capabilities • User documentation	• Limited feature set • ITIS hub dependency • Partial integration	• Integration challenges • User training needs • Mobile device access	*“The End TB app suite … is also linked with the main system which is the ITIS. So actually ITIS acts as the hub for these different technologies.”*
	DataToCare	• Automated lab results • No manual data entry • GeneXpert data extraction • Faster result delivery	• GeneXpert computer needed • Technical setup required • Subscription cost	• Technical integration • Lab staff training • Equipment needs	*“Since they are installed in the GeneXpert in the computer, they can extract the data from the GeneXpert machine and then push the laboratory data to ITIS so that the medical technologies will not have to manually encode the results.”*
Tanzania	ETL (DHIS2)	• Flexible customization • Local development • MOH system integration • Easy data analysis	• No initial offline mode • Internet dependency • District-level limitation	• Internet connectivity • Computer literacy gaps • Hardware constraints	*“The flexibility it has. I think it is wonderful whereby you can really customize to your own environment in your own context.”* *“The flexibility it has. I think it is wonderful whereby you can really customize to your own environment in your own context.”*
	DHIS2 Android App	• Offline collection potential • Mobile accessibility • Reduced coordinator need	• Offline mode in testing • Android device requirement	• Mobile hardware needs • Training requirements • Synchronization issues	*“They’re telling us this one can be offline mode, can operate as an offline.”*
	Tambua TB	• SMS-based access • Facility referral system • Basic phone compatibility • Low access barriers	• Screening function only • Limited option flow	• User awareness • Phone access • Follow-up challenges	*“It is a platform where somebody can send a text message in that special number and then questions will be coming … if they are likely to have symptoms of TB then they will be advised to go to the nearest facility for further investigation.”*
Vietnam	VITIMES (2013)	• Central TB database • Simplified reporting • Improved data accuracy • National implementation	• No offline capability • Computer requirement • Complex interface • Paper backup needed	• Limited rural infrastructure • Aging hardware • Connectivity issues • Staff technology barriers	*“So for the patient management system, the national TB program, they have the big system to manage TB patient started from 2013 and just updated it to the newer version this year.”*
	New VITIMES (2023)	• Enhanced modules (latent/MDR-TB) • Updated interface • Comprehensive data collection	• More complex system • Overwhelming for commune staff • Higher technical demands	• Intensive module training • Limited training capacity • Advanced tech knowledge	*“Right now they are merging everything into one big system. And for the regular staff at the commune level they can be overwhelming of the things that they see on one big system.”*
	ACIS	• Offline mode • Mobile compatibility • Flexible implementation • Combined screening/management	• Optional facility use • Limited adoption • Parallel VITIMES system	• Limited implementation areas • Training requirements • VITIMES integration issues	*“ACIS, in the past was developed by Clinton Health Access Initiative and they gave it to, they share to NTP to use for the NTP active case filing.”*

### Appendix C

**Table:** 

Sub Themes	CFIR Subdomains	Quotes
**I. Cfir Innovation Domain** **Theme: Characteristics of an effective TB information system**		
International collaboration serves as a crucial evidence review, creating awareness of global technologies and enabling their adaptation to local context.	Evidence-Base	“We have so many TB projects (from development partners) … from the start of late 90’s, the first global fund project coming into this nation and it was followed by the USAID … and other partners … so some of the practice they brought from the other countries.” ~ T1002, Indonesia
		“… we have some experts in Indonesia especially in TB and some of them already been … exposed to the other best practice, especially technology for the TB program itself …. we have recommendation from the expert itself. “~ T1002, Indonesia
		“this is a similar model as of Nikshay. So we got support from Gates Foundation and they supported us with the concept paper and … the details of how the call center and how Nikshay is working in India” – T1011, Pakistan.
		“We had some pilot experience on initial small initiative and based on the learnings we expand and scale it up in no time.” ~ T1009, India.
		“So DHIS2 is used worldwide and there are plenty of examples from different countries that was reviewed and then adapted.” ~ T1011, Pakistan.
Enhanced data quality offers detailed patient-level information, prioritizing patient-centric care over aggregate data.	Relative Advantage	“The purpose of TIBU lite is to facilitate patient level data capture within the private settings” ~ T1005, Kenya.
		“India introduced the case-based surveillance system called Nikshay and that was … a major change in terms of the program management” ~ T1009, India.
		“There were issues in manual data collection, there could be errors both in terms of data entry and transcription.” ~ T1011, Pakistan.
		“The number of TB patient notified is not correct, because sometimes they forgot to enter something coming from the paper-based into VITIMES”~T1004, Vietnam.
		“The absence of the digital tools, … can delay the data collection process, leading to outdated information and hindering the decision making processes.”~ T1011, Pakistan.
		“There is still recording on the paper ongoing and this is dual work and extra responsibility on the staff” ~ T1009, India.
		“I’ll say as a strength is one the quality improvement of the data, the accuracy has improved.” ~ T1011, Pakistan.
		“So advantages I would say is that when I compare with other verticals in health … all the information needed is there in the application.”~ T1010, India.
Access to data extends beyond NTP through increased user authorization and streamlined data sharing initiatives.	Design	“(In) paper-based records, there could be data-security or privacy related issues.” ~ T1011, Pakistan.
	Relative Advantage	“If the Janao app is well used by all the practitioners who are dealing these patients so I think it’s much easier to get those notifications from practitioners”.~ T1006, Bangladesh.
		“The users were having, based on their email id, access and only … the authorized users can enter the data and edit the data at the higher level.” ~ T1009, India.
		“Sharing of that data … has really helped with data digitalization” ~ T1011, Pakistan.
Real-time reporting and simplified analysis facilitate efficient tracking, reporting, and decision-making in TB care	Design	“for the facility that has no Internet connection it will be a challenge … So ITIS has desktop or offline version and then it also has a online or web version to cater to both scenario.” ~ T1012, Philippines.
	Complexity	“… for the health personnel using the WIFI TB … Some of the GPs saying that this is easy to use because it is simple, and mobile, and … it doesn’t have the … buffering process like the SITB … So it’s easy to use.” ~ T1002, Indonesia.
	Relative Advantage	“I think other … benefits … that ITIS has provided is the … timeliness of the report … since there’s ITIS … the central office already has a real time data from the facility …” ~ T1012, Philippines.
		“… your manual data collection tool did not support real-time reporting.”~ T1011, Pakistan.
		“the biggest advantage is reducing the workload of the of the healthcare staff at the site level”~ T1004, Vietnam.
		“So (the advantage was) first of all we can record and report in a timely manner” ~ T1002, Indonesia.
		“All they need to do is to generate the report by clicking the the generate button and then submit it officially to the National TB program.”~ T1012, Philippines.
		“So for the … TB program improvement based on those data … the information system is really helpful for us to make the data set and … for the better program implementation.” ~ T1002, Indonesia.
		“… the DHIS2 tracker was … only providing … aggregated data … it will only tell like from this district or this province, these many cases are notified. We will not be able to follow up with those cases or we will not be able to make any real-time decisions based on that data …” ~ T1011, Pakistan.
		“… since we have developed the IT slide, we have developed a referral module so that the private physician can still follow up what happened to the patient that he or she referred to the public facility.” ~ T1010, India.
		“… there are registers which were clinic-based registers and then district-based registers … all of these registers were manually filled to make sure that the information of each and every patient is there who gets registered … there’s … still a lot of paper-based work …” ~ T1011, Pakistan.
The capacity to integrate with additional apps, and be compatible with mobile phones, enhances their overall utility.	Design	“We have also created a mobile app for the ITIS.” ~T1012, Philippines.
	Adaptability	“… there are different … digital tools that are linked with ITIS … we have the NTB app suite … mobile apps for dashboard, for screening tool … for users manual and … monitoring and evaluation guide … ITIS acts as the hub for these different technologies.” ~ T1012, Philippines.
		“… there is a hospital … they have their own EMR and … they are recording their patients. So we have created an API that will receive data from these private hospitals.” ~T1012, Philippines.
		“… the most important thing was the patient support system which was possible … only with … a digital system like the case-based system of Nikshay … When call support is linked with the Nikshay there is nothing like that and we can really move ahead with the implementation of this direct benefit transfer wherein the bank accounts details are seeded …” ~T1009, India.
		“… patient support system, direct benefit transfers … there are several linkages with the support system, including the nutritional support … digital adherence system, all those things were not possible before which are there with the patient centric approach …” ~T1009, India.
The newer systems are not foolproof and have technological limitations that could benefit from improvement.	Design	“… the automated SMSs on all levels in different languages to the patients and to the providers were a regular feature of Nikshay in the beginning to help in improving and scaling it up, but for whatever changes have happened, we have lost track of those SMS’s for few years.” ~ T1009, India.
		“… now we are collecting the data but the data is too big. So … especially during reporting period we are experiencing some server slow down in the system.” ~ T1012, Philippines.
		“… disadvantages come with the non-availability of internet at times … we ensure that the service is not denied to the individual and … they get similar service but because of non-availability of internet sometimes there is a delay …” ~ T1009, India
	Complexity	“I used to visit a few practitioners and also had the chance to discuss with them about the app … they praised about it but the … problem is they have really little time to enter those datas in the app … they find that it’s a little bit time consuming so they are reluctant to do that.” ~ T1006, Bangladesh.
The cost for systems is usually covered by the NTP. How much does it cost the NTP?	Cost	“… almost 30,000 tablets have been procured and supplied to the health facilities … the data component … is compensated by the program through NHM on a regular basis … you get almost unlimited data packs in the country … So really in that way there is no cost …” ~ T1009, India.
		“… there is no cost for the user. Because for DHIS2 tracker application, it is being used by our field officers who are … staff of the project … we have provided them with the tablets through the project funds that was part of the initial budget as well.” ~ T1011, Pakistan.
**II. Cfir Outer Setting Domain** **Theme: Contextual factors in implementing the technology**		
Local conditions impact the implementation of technology	Local Conditions	“… the regular supplies of drugs and diagnostics was the hallmark of the program over years. But last two years, we are … witnessing disruptions deep in the reserve stocks of the drugs and diagnostics … That is the biggest weakness I can see right now on the program.” ~ T1009, India.
		“… we are facing a lot of challenges in terms of the change in the management of the national and provincial TB program. That’s mainly because of … the change in … political scenarios in the country.” ~T1011, Pakistan.
Policies mandating notification and exclusive reporting on the new system improve their uptake.	Policies & Laws	“Yeah there is a government passed a law like every TB case should be notified. There is always like that.” ~T1006, Bangladesh.
		“Right now because there was … a memo from the central office of the Department of Health that … they only have to use the ITIS for the TB recording and the reporting.” ~T1012, Philippines.
**III. Cfir Inner Setting Domain** **Theme: Challenges and opportunities within the NTPs**		
There’s a significant dependence on international funding, contrasting with limited collaboration within countries	Relational Connections	“… there’s a sizable proportion of staff that are donor fund … if donor funding stops then it may hamper how our service delivery is done … the program itself has a significant amount of donor reliance … as opposed to domestic resourcing …” ~ T1005, Kenya.
		” … the TB problem … should be managed beyond health itself … TB works is still on the health sector itself … it goes with the utilization of the district funding … it doesn’t utilize for the TB program elimination program … So, we still lack … coordination with the sector beyond health right now.” ~ T1002, Indonesia.
Newer systems require updates to existing policies and systems for compatability	Structural Characteristics	“… when we shift from the paper-based to the digital system … we also need to … revise our systems and policies with regards to the data validation … every quarter … we pay them a small incentive against each registered patient … that could be a potential challenge where people who are used to … implement a certain system, are now shift to another …” ~ T1011, Pakistan.
Infrastructure and training needs were consistent across countries, with no significant variations.	Information Technology Infrastructure	“… most of the state-owned use desktops. So desktop is outdated especially at remote area.” ~ T1004, Vietnam.
		“… these apps needs internet in some parts of the country there is very limited internet access so this is really a challenge.” ~ T1006, Bangladesh.
		“… WIFI TB … this the mobile application which we can download it on the IOS system or from google play store and it is for the general practitioners.” ~ T1002, Indonesia.
		“… initially we started with our own physical servers and a team … developing Nikshay. Later on we shifted to the cloud based services and expanded based on the requirement as and when we scale it up.” ~ T1009, India.
		“… the field staff, they have been provided with the … tablets and they are using those tablets for entering the data on the DHIS2 tracker application for the providers, they just have to notify.” ~ T1011, Pakistan
		” … for the direct cost … it’s quite big … because we need to have a big server to ensure that we have so many health facilities in the country.” ~ T1002
	Work Infrastructure	“… as we know especially in the private sector … the turnover of stuff is quite high … So we need extra effort to continuously train the health facility for using this kind of information system.” ~ T1002, Indonesia
The NTP has several strengths that serve as robust foundations for implementing and integrating new information systems.	Work Infrastructure	“… our TB program is well structured, very well established … and based on that it has a very good technical capacity in different thematic areas in relation to TB.” ~ T1005, Kenya.
		“… the structure is very well clearly understood from the national level all the way to the county to the sub county level as well as to the health facility level. There’s very clear demarcation of the roles and responsibilities.” ~ T1005, Kenya.
	Relational Connections	“There are some main supports … from the government itself or from the … development partner and also from the … UN agencies, WHO, UNICEF, and USAID. So we have so many supports.” ~ T1002, Indonesia.
		“… the contribution of the private sector has drastically improved … whenever there is a … grant making process or a strategy development process, both public and private sector … are engaged and … they jointly develop … strategies and policies …” ~ TI1011, Pakistan.
	Mission Alignment	“… I would say that with the passage of time … technology has been incorporated in the TB program like the use of expert testing machines, the other diagnostic tools like X-ray machines …” ~ T1011, Pakistan.
	Culture	“… I would say that there are technical people in the national provincial TB control program who are … steering this program and who are really, really committed to the program.” ~ T1011, Pakistan.
The limitations of previous systems necessitated urgent change, leading to the adoption of new systems.	Tension for Change	“… the challenge … with the DHIS2 tracker was it was only providing … aggregated data … it will only tell … from this district or this province, these many cases are notified. We will not be able to follow up with those cases or … make any real-time decisions based on that data.” ~ T1011, Pakistan.
		“… the absence of … digital tools, they require the healthcare providers to manually record and document … TB related data … which is a very time-consuming process which … often … can delay the data collection …” ~ T1011, Pakistan.
		“… in the … previous aggregated reporting it was mainly … quarterly … reporting by the … districts and … states … and … it would take around six months … for the action to be taken by the higher level … So you can say there was a delay in overall recording, reporting, and action … ~ T1009, India.
		“… all the piles of the paper-based records … make it difficult to analyze the trends or monitor the progress.” ~ T1011, Pakistan.
		“… the absence of digital tools … makes it difficult to share data and information efficiently with different stakeholders which has been one of the challenges between the public and private sector in the country.” ~ T1011, Pakistan.
		” … in the WIFI TB, the mobile application for reporting and recording the TB cases … we see that the general practitioners is not really … likes the SITB system because it is too complicated its … exhausted to input all of those variables.” ~ T1002, Indonesia.
Certain NTPs employ incentives to drive adoption of new information systems.	Incentive Systems	“But before we are … giving awards like certificates to best performing area for ITIS.” ~ T1012, Philippines.
		“… if you are only … talk about the PPM Hub and the DHIS2 tracker, there are no … financial incentives … other than … capacity building activities and the provision of the internet facility …” ~ T1011, Pakistan.
		“… 1000 rupees for each patient. 500 for notification and 500 on treatment completion … there are financial and non-financial incentives for better performance which is dependent on Nikshay entries.” ~ T1009, India.
Training and support services are readily available to assist with the transition to the new system.	Access to Knowledge & Information	“For TBCP … they have the trainings both physically and online they have … guidelines. The guidelines are uploaded in the NTP’s websites. They can download it from the NTP’s website and use it whenever they need.” ~ T1006, Bangladesh.
		“… we have the video tutorial video for using SITB … the technical guideline and … videos … uploaded in youtube as well as the technical guideline … stored in the … online drive. So we can quickly access the link and also download it.” ~ T1002, Indonesia.
		“… during the transition period from ETR and e-TB manager to ITIS, we coordinated with the developers … So this database were converted to be compatible with the structure of the ITIS. So all of the data … from the ETR and e-TB manager are in ITIS right now.” ~ T1012, Philippines.
**IV. Cfir Individuals Domain** **Theme: Influence of personal attributes on technology**		
Increased Comfort with Technology in modern days simplifies the use of these systems	Implementation Facilitators	“… the more you are knowledgeable on the technology … the higher you’re able to navigate through the technology.” ~ T1001, Tanzania.
		“… people are very well-versed nowadays, not just the younger generations but even the older generation in India you would see them on WhatsApp and Facebook all the time.” ~ T1009, India.
**V. Cfir Implementation Process Domain** **Theme: Implementation challenges and strategies**		
The attitude of people toward change significantly impacts their ability to fully embrace it	Adapting	“… people who are using paper-based systems for many years they are not used to … implement these digital technologies … Some of them thought that … continuing with old practice will be easier … they are entering the data both in the application … and in the hard form, this has … doubled their work.” ~ T1011, Pakistan.
	Engaging	” … regarding the mobile application like the Janao app … not all the practitioners … use this app. So, we are not getting that much of data from that application.” ~ T1006, Bangladesh
	Adapting	“It is not the difficulty in using Nikshay, it is the reluctance sometimes.” ~ T1009, India.
Efficient implementation processes is facilitated by effective strategies	Teaming	“… in government health facilities … they start from 8.30 in the morning … to 2.30. So it is challenging for the laboratory technicians … putting all the data regularly. So … we start our operation from 9 up to 6 pm. So we have a longer time … to ensure data entry.” ~ T1006, Bangladesh.
	Assessing Needs	“For a new technology, first we are doing … requirements analysis and then … gathering all … stakeholders … involved … for a specific system and then gather their requirements and then consult with … who will be the system owner …” ~ T1012, Philippines.
	Tailoring Strategies	“… once they have the buy in … see the advantage … the process starts as to … what should it look like … who should develop it … and then … an in house capacity … including the NICM and who were supposed to give the technical inputs and support to the program …” ~ T1009, India.
	Access to Knowledge & Information	“… during the pilot period of ITIS we have conducted the training … for pilot sites.” ~ T1012, Philippines
	Reflecting & Evaluating	” … focus group discussions, regular focus group discussions with the … users … continuous feedback was inbuilt in the … program … that we should collect regular feedback from the users so that the application should be made as much user-friendly as possible.” ~ T1011, Pakistan.
	Reflecting & Evaluating	“… every two years, we are conducting a users conference. We are inviting different users from different area and then also from the private sector. So far they are happy with the ITIS.” ~ T1012, Philippines

## Electronic supplementary material

Below is the link to the electronic supplementary material.

Supplementary Material 1

## Data Availability

The datasets generated and/or analysed during the current study are not publicly available due but are available from the corresponding author on reasonable request.
