# Supplementary material for: Implementation of digital tuberculosis information systems: perspectives from 10 high TB burden countries
Source: BMC Infect Dis. 2026 Apr 2;26:1030. doi: 10.1186/s12879-026-12852-3 (PMC13202920; doi:10.1186/s12879-026-12852-3)
Supplement: Supplementary file 1 — Supplementary Material 1 [file 12879_2026_12852_MOESM1_ESM.pdf]

Interview Guide: Healthcare Provider Perspectives on Technology Use in TB Care Cascade

Demographic

1. **What is your age group?**
    - *<25,*
    - *25 to 35,*
    - *35 to 50*
    - *>50*
2. **What is your highest level of education/certificate/degree?**
   MPH

Introduction

3. **What is your role in TB prevention and care?**
    - *Probe: the position /role; what you actually do; what are your defined responsibilities: how long have you worked* at your current organization
4. ***What do you think of overall TB management in your country?***
    - *Probe: Strengths and weaknesses of current TB program in your country*

5. **What role does technology play in TB prevention and care in your country?**
6. **Name some of the technologies that are prevalent in your country.**

   (For each of the technologies mentioned, go through the questions below)

Intervention Characteristics

Name of the technology: ____________________________

Intervention Source

- Who developed the Technology?

Evidence Strength & Quality

- Is there evidence about the use of this technology?
    - What evidence have you heard about from your own research? Practice guidelines? Published literature? Co-workers? Other settings?

Relative Advantage

- How does the technology compare to other similar existing programs in your country?
    - What advantages does the technology have compared to existing programs?
    - What disadvantages does the technology have compared to existing programs?
    - Is there an alternative that you would rather implement?

Adaptability

- What kinds of changes or alterations do you think you will need to make to the intervention so it will work effectively in your country?
- Are there components that should not be altered?

Complexity

- How complicated is the technology?

Design Quality & Packaging

- Are you aware of any online resources, marketing materials, or a toolkit, to help with training for the technology.

Cost

- Does the technology involve any cost? Which are direct and indirect costs?

Outer Setting

Patient Needs & Resources

- Have you elicited information from recipients regarding their experiences with the technology?
  - What are their perceptions of the intervention?
  - Can you describe what kind of specific information you have heard?

Peer Pressure

- What are the organizations in your country that are implementing this technology? To what extent?

External Policies & Incentives

- Name some key stakeholders important in the implementation of technology.

- What kind of local, state, or national performance measures, policies, regulations, or guidelines influences the decision to implement the technology?

- Are there any financial or other incentives that could influence the decision to implement the intervention?

Inner Setting

Structural Characteristics

- What kinds of infrastructure changes is needed to accommodate the technology?

Compatibility

- Before the technology, how did organizations carry out the functions?

- Can you describe how the technology was integrated into or replaced the existing processes?
  - How did it interact or conflict with current programs or processes?

Individual Characteristics

Personal Attributes

- How does a personal ability to use the technology influence the effectiveness of the technology?
  - Have you noticed anybody within your organization struggle to use this technology? Why?

Implementation process

- What do you think would be needed to implement the technology in a newer organization in your country? What is the process they must follow?

Additional Questions

1. Do you use this technology for TB services in your organization?
    - Yes
    - No
- How often do you use the technology?
    - More than once a day
    - Once a day
    - Once a week -
    - Once a month -
- How has the technology contributed to your outcome/indicator (e.g. Notification, Detection)?
    - Increase – What % over what period
    - Decrease– What % over what period

- Do you use the technology for all people? What are some exemptions? How many on an average?

Can you suggest a few stakeholder who will be vital to be interviewed for next round?
